# Associations of Plasma Lipidomic Profiles with Uric Acid and Hyperuricemia Risk in Middle-Aged and Elderly Chinese

**DOI:** 10.1007/s43657-024-00157-x

**Published:** 2024-07-30

**Authors:** Wanhui Kang, Xinming Xu, Xiaowei Yang, Qingqing Wu, Shuning Li, Keran Gao, Rong Zeng, Liang Sun, Xu Lin

**Affiliations:** 1 Key Laboratory of Systems Health Science of Zhejiang Province, School of Life Science, Hangzhou Institute for Advanced Study, University of Chinese Academy of Sciences, 1 Xiangshanzhi Ln., Hangzhou, 310024 China; 2 Department of Nutrition and Food Hygiene, School of Public Health, Institute of Nutrition, Fudan University, 130 Dongan Rd., Shanghai, 200032 China; 3 Shanghai Institute of Nutrition and Health, Chinese Academy of Sciences, 320 Yue-Yang Rd., Shanghai, 200031 China; 4 Shanghai Institute of Biochemistry and Cell Biology, Center for Excellence in Molecular Cell Science, Chinese Academy of Sciences, 320 Yue‑Yang Rd., Shanghai, 200031 China; 5 Schulich School of Medicine and Dentistry, Western University, 1465 Richmond St, London, ON N6G 2M1 Canada

**Keywords:** Hyperuricemia, Chinese, Lipidomic, Uric acid

## Abstract

Little is known about the links of disturbed lipid metabolism with hyperuricemia (HUA). We aimed to investigate the associations of lipidomic profiles with uric acid (UA)/HUA and their modifying factors in middle-aged and elderly Chinese. A total of 350 lipids were quantified in 2247 community-based Chinese aged 50–70 years by high-coverage targeted lipidomics. HUA was defined by plasma UA > 420 μmol/L in men or > 360 μmol/L in women. The prevalence of HUA in this population was 10.4%. After multivariable adjustment including BMI and lifestyle, 123 lipids were significantly associated with UA, predominantly glycerolipids (GLs) and glycerophospholipids (GPs). Specifically, diacylglycerol [DAG (16:0/22:5), DAG (16:0/22:6), DAG (18:1/20:5), DAG (18:1/22:6)], phosphatidylcholine [PC (16:0/20:5)), and triacylglycerol (TAG (53:0)] were the most significant lipid signatures positively associated with HUA risk, while lysophosphatidylcholine (LPC (20:2)) was inversely associated with HUA risk (*p* < 0.05). Network analysis also showed a positive association between TAGs/PCs/DAGs contained module and HUA risk (*p* < 0.01). Notably, HUA-related lipids were associated with de novo lipogenesis fatty acids, especially 16:1n-7 (Spearman correlation coefficients = 0.32–0.41, *p* < 0.001). Reduced rank regression showed that increased aquatic products intake was correlated to elevated HUA risk and HUA-associated lipids; while high dairy consumption was correlated with low level of HUA-associated lipids (|factor loadings| ≥ 0.2). Moreover, mediation analyses suggested that the lipid-HUA associations were partially mediated by retinol-binding protein 4 (RBP4, mediation proportion 5–14%), an adipokine linked with dyslipidemia and insulin resistance. In conclusion, disturbed specific metabolisms of GLs and GPs were associated with high prevalent HUA, partially mediated by RBP4 and/or influenced by certain dietary factors.

**Supplementary Information:**

The online version contains supplementary material available at 10.1007/s43657-024-00157-x.

## Introduction

Hyperuricemia (HUA) reflects perturbed purine metabolism characterized by elevated blood uric acid (UA) concentrations. HUA has been a growing global health issue with a prevalence reaching 14% among Chinese adults during 2018–2019 (Zhang et al. 2021) and approximately 20% in the United States (2015–2016) (Chen-Xu et al. 2019). Uric acid is regulated through a homeostatic balance between dietary intake or endogenously yielded by purine metabolism and excreted by the intestine (~ 30%) and kidneys (~ 70%) (Mandal and Mount 2015). HUA is not only attributed to inflammatory arthritis or kidney stones by depositing urate crystals in various tissues and organs, but also increases the risks of renal dysfunction, metabolic syndrome, type 2 diabetes, and cardiovascular diseases (de Magalhaes et al. 2021; Wu et al. 2021). Therefore, understanding the etiology and factors involved in the physiopathogenesis of HUA is essential for preventing these diseases.

Dyslipidemia such as elevated circulating total triglycerides and low-density lipoprotein cholesterol (LDL-C) were suggested to be associated with raised UA in some population-based studies (Liang et al. 2020). However, these conventional clinical lipid measurements are unable to provide detailed insights into specific perturbed lipid metabolism. Lipidomics, on the other hand, enables to comprehensive reveal a wide range of lipid subclasses with diverse structures, which offers opportunities to underline potential metabolic pathways and related health conditions (Fahy et al. 2011; Hornburg et al. 2023). Nevertheless, few large-scale epidemiologic studies, if any, have investigated the relationship between different lipidomic classes and UA/HUA so far.

Circulating lipidomic profiles could be influenced by endogenous and exogenous factors. Previous studies in our and others showed the effects of certain dietary factors with specific lipids (Chen et al. 2021; Floegel et al. 2013; Liu et al. 2022; Quehenberger et al. 2010; Yun et al. 2022). For instance, we found that the diabetes-associated phosphatidylcholines (PCs 16:0/16:1, 16:0/18:1 and 18:0/16:1) and phosphatidylethanolamine (PE 16:0/16:1) could be influenced by fatty acids (FAs) in the de novo lipogenesis (DNL) pathway and also diets containing high refined grains and low fish, dairy and soy products in the same study population as the current study. On the other hand, UA metabolism per se could be affected by dietary factors like seafood, meat, as well as dairy products (Choi et al. 2004, 2005). Therefore, it is interesting to know the effects of dietary factors on lipidomic profile and/or HUA risk.

Thus, using high-coverage targeted lipidomics, we aimed to investigate the associations of lipidomic profiles/subclasses with plasma UA levels or with HUA risk and to examine the modifying factors on the associations in a well-characterized Chinese cohort.

## Materials and Methods

### Study Population

The study population was from the Nutrition and Health of Aging Population in China (NHAPC) study, which aimed to investigate the effects of genetic and environmental factors on cardiometabolic diseases. The detail of the study was described elsewhere (Ye et al. 2007). In brief, 3289 community-dwelling residents aged 50–70 years were recruited from both rural and urban areas in Beijing and Shanghai in 2005. This study was approved by the Institutional Review Board of the Institute for Nutritional Sciences, Chinese Academy of Sciences (E-2005-01). Each participant was provided written informed consent. After excluding 1041 individuals without lipidomic and one participant without UA data, a total of 2247 participants were included in the current analyses. The overall study process and analysis plan are presented in Fig. S1.

### Data Collection

A face-to-face interview was conducted to collect information on demographics, lifestyle, health, and medication status using a standardized questionnaire by well-trained health professionals. Education level was classified according to self-reported school years (0–6, 7–9, and ≥ 10 years). Physical activity was categorized as low, moderate, or high according to the sum of the metabolic equivalent score derived from the short form of the International Physical Activity Questionnaire (Craig et al. 2003). A family history of chronic diseases was defined as if a parent or first-degree sibling had hypertension, diabetes, or cardiovascular disease (Ye et al. 2007). Hypertension was defined as systolic blood pressure (SBP) > 140 mmHg, diastolic blood pressure (DBP) > 90 mmHg, taking antihypertensive medications, or self-reported physician diagnosis (Wang et al. 2018). A 74-item food frequency questionnaire, modified from the validated questionnaire used in the 2002 National Nutrition and Health Survey in China (Zhao et al. 2002), was employed to determine food intake frequency and amount in the past year before the study. Main food items (g/day) were categorized into 15 groups for further analyses (Table S1).

All participants were invited to undertake a physical examination after overnight fasting. Anthropometric measurements such as weight, height, waist circumference (WC), and blood pressure were performed following a standard protocol (Ye et al. 2007). Body mass index (BMI) was calculated as weight divided by square of height (kg/m^2^). Fasting venous blood samples were collected in tubes containing ethylenediaminetetraacetic acid, centrifuged at 3000 rpm for 15 min at 4 °C aliquoted into cryovials, and stored at − 80 °C until analysis.

### Laboratory Measurements

Plasma fasting glucose, insulin, total cholesterol (TCH), high-density lipoprotein cholesterol (HDL-C), LDL-C, total triglycerides, *γ*-glutamyl transpeptidase (GGT), alanine aminotransferase (ALT), aspartate transaminase (AST), creatinine, urea nitrogen, and UA were measured by an automatic analyzer (Hitachi 7080) using commercial kits from Wako Pure Chemical Industries. Retinol binding protein 4 (RBP4) was measured by a sandwich enzyme-linked immunosorbent assay (ELISA) developed in-house as previously detailed (Qi et al. 2007). Homeostatic model assessment of insulin resistance (HOMA-IR) was calculated as fasting glucose (mmol/L) × fasting insulin (μU/mL)/22.5. Estimated glomerular filtration rate (eGFR) was calculated using the Modification of Diet in Renal Disease study equation for Chinese: eGFR (mL/min per 1.73 m^2^) = 175 × creatinine (mg/dL)^−1.234^ (Jaffe's kinetic method) × age^−0.179^ (× 0.79 if female) (Kong et al. 2013). Declined kidney function was defined as having eGFR < 60 mL/min per 1.73 m^2^ (Levey et al. 2011). Erythrocyte FAs were measured by gas chromatography coupled with positive chemical ionization (Agilent 6890 N-5975B; Agilent Technologies, USA) (Zhang et al. 2012).

### Outcome Ascertainment

HUA was defined as plasma UA > 420 μmol/L (7.0 mg/dL) in men and > 360 μmol/L (6.0 mg/dL) in women according to the Chinese Multidisciplinary Expert Consensus on the Diagnosis and Treatment of Hyperuricemia and Related Diseases (Multidisciplinary Expert Task Force on Hyperuricemia and Related Diseases 2017).

### Lipidomic Profiling

Plasma lipidomic profiles were quantified by liquid chromatography–electrospray ionization mass spectrometry. Details on lipid extraction, chromatographic separation, mass spectrometry analysis, and data quantification were provided elsewhere (Yun et al. 2020). Briefly, lipids were extracted following a modified methyl tert-butyl ether protocol and then analyzed on a Shimadzu Nexera X2 LC-30AD system coupled with a SCIEX 5500 QTRAP mass spectrometer. Analyst 1.6.3 software (Sciex, Foster City, CA) was used for data acquisition. Plasma samples were analyzed in random order and quality control samples were inserted every 10 samples to ensure repeatability. After excluding those with missing data > 20% or the coefficient of variation > 30%, 728 valid lipids were quantified, including four main classes: glycerophospholipids (GPs) [54 PCs, 10 lysophosphatidylcholines (LPCs), 92 PEs, three phosphatidylserines (PSs), one phosphatidylinositol], sphingolipids (SPs) [33 ceramides, 43 sphingomyelins (SMs)], sterol lipids [10 cholesterol esters (CEs)], and glycerolipids (GLs) [25 diacylglycerols (DAGs), 79 triacylglycerols (TAGs), and 378 TAG fractions]. Missing values for lipids were imputed as half of the minimum values given that the concentrations might be under the lower detection limits. After further integrating TAG fractions, 350 lipids were included in the final analyses.

### Statistical Analyses

Differences in characteristics between HUA and normouricemic populations were evaluated by the Student’s *t*-test or Wilcoxon’s signed rank test for continuous variables, while chi-squared test were used for categorical variables. Concentrations (mg/L) of 350 lipid metabolites were naturally log-transformed if the absolute skewness was over one and then standardized to the normal distribution (mean = zero, standard deviation = one) before analyses. The lipid metabolites were considered as significantly different after multiple testing by the false discovery rate (FDR) between participants with and without HUA (Benjamini and Hochberg 1995). Spearman correlation coefficient (*r*_s_) were calculated to evaluate inter-correlations among the 350 lipids, or between lipids and cardiometabolic traits. Associations of lipids with UA concentrations and HUA risks were assessed by multivariable linear regression and log-Poisson regression models, respectively, adjusting for demographic [sex, age, region (north or south), residence (urban or rural), education attainment] and lifestyle characteristics (smoking status, drinking status, physical activity), cardiometabolic traits (BMI, GGT, creatinine, HOMA-IR, total triglycerides, and TCH), hypertension, family history of chronic diseases and medication status (antihypertensive and lipid-lowering medicines). The risk of HUA was evaluated using risk ratios (RRs) with a 95% confidence interval (CI). The significance of each lipidomic signature was corrected by the FDR correction. Mediation analyses were performed to investigate the potential mediator impacting the individual lipid-HUA relationship adjusting for the aforementioned covariates. The variances of mediation effects were calculated by the quasi-Bayesian Monte Carlo method with 100 simulations. Sensitivity analyses were conducted to test the robustness of findings by excluding participants with lipid-lowering medication or declined kidney functions. Stratified analyses were performed according to age, sex, region, residence, smoking status, drinking status, hypertension, type 2 diabetes, self-reported cardiovascular diseases (stroke and ischemic heart disease), medication uses, BMI, GGT, creatinine, HOMA-IR, total triglycerides, and TCH. Likelihood ratio tests were utilized to evaluate the statistical significance of effect modification (*p-*value for interaction). The models with and without interaction terms between lipid species and the effect modifiers were compared.

Weighted gene co-expression network analysis (WGCNA) was performed to explore biological networks and identify modules consisting of highly correlated lipids. To assess the correlation between pairs of lipids, a Pearson correlation matrix was calculated and then weighted by a soft-thresholding power of seven, based on the scale-free topology criterion (Fig. S2). This process generated an adjacency matrix, which was transformed into a topological overlap matrix to measure dissimilarity. The resulting dissimilarity was utilized for hierarchical clustering, generating a dendrogram that grouped correlated lipid species into distinct branches. The dynamic tree-cut algorithm was then employed to identify modules containing interconnected branch nodes, with a minimum size requirement for module detection of 10. For each module, a representative value called the “eigenlipid” was computed. “Module membership” indicated the correlations of a specific lipid with the module, while “lipid significance” indicated the associations between the lipids and HUA risk. Furthermore, a network heatmap of the topological matrix was generated, and an unsigned node-edge network plot was created using Gephi (version 0.9.2) for visualization purposes. Associations of WGCNA modules with UA concentrations and HUA risks were assessed by multivariable linear regression and log-Poisson regression models, respectively, adjusting for the aforementioned covariates.

Reduced rank regression (RRR) was applied to estimate the associations between food groups and plasma lipidomic biomarkers. The RRR approach identifies a series of predictors (e.g., food groups) to maximumly explain variations in responses (e.g., lipids) (Hoffmann et al. 2004; Schulze et al. 2003). After adjusting for age, sex, region, and residence, dietary factors were identified for the lipids that were significantly associated with HUA using 15 predefined food groups (Table S1). Food groups with absolute loading values > 0.2 were considered prominent (Schulze et al. 2003). All analyses were conducted by R 4.1.3 (R Foundation for Statistical Computing). A two-sided *p*-value < 0.05 was considered statistically significant unless specified.

## Results

### Characteristics of Participants

The prevalence of HUA was 10.4% (234 of 2247). Compared with normouricemic counterparts, participants with HUA were more likely to be older and to reside in southern and rural areas. They also exhibited unfavorable cardiometabolic traits, including higher levels of BMI, DBP, insulin, HOMA-IR, total triglycerides, TCH, LDL-C, and lower HDL-C (*p* < 0.05), as well as higher concentrations of ALT, AST, GGT, creatinine, and urea nitrogen (*p* < 0.05) (Table 1).

**Table 1:** Characteristics of participants between those with and without hyperuricemia

Characteristics	Total (*n* = 2247)	Hyperuricemia		*p*-value
		No (*n* = 2013)	Yes (*n* = 234)	
Age, years	58.2 (5.95)	58.1 (5.91)	59.7 (6.07)	**< 0.001**
Female, *n* (%)	1297 (57.7%)	1179 (58.6%)	118 (50.4%)	**0.021**
Northern residents, *n* (%)	1114 (49.6%)	1024 (50.9%)	90 (38.5%)	**< 0.001**
Rural residents, *n* (%)	964 (42.9%)	821 (40.8%)	143 (61.1%)	**< 0.001**
Education level, *n* (%)				**< 0.001**
0–6 years	1024 (45.6%)	935 (46.4%)	89 (38.0%)	
7–9 years	782 (34.8%)	710 (35.3%)	72 (30.8%)	
≥ 10 years	441 (19.6%)	368 (18.3%)	73 (31.2%)	
Current smoker, *n* (%)	614 (27.3%)	551 (27.4%)	63 (26.9%)	0.950
Current drinker, *n* (%)	562 (25.0%)	505 (25.1%)	57 (24.4%)	0.870
Physical activity, *n* (%)				**< 0.001**
Low	153 (6.81%)	137 (6.81%)	16 (6.84%)	
Medium	880 (39.2%)	759 (37.7%)	121 (51.7%)	
High	1214 (54.0%)	1117 (55.5%)	97 (41.5%)	
Medication status, *n* (%)				
Lipid-lowering medication	141 (6.28%)	115 (5.71%)	26 (11.1%)	**0.002**
Antihypertensive medication	590 (26.3%)	481 (23.9%)	109 (46.6%)	**< 0.001**
Family history of chronic diseases	1226 (54.6%)	1074 (53.4%)	152 (65.2%)	**0.001**
SBP, mmHg	140 (22.4)	140 (22.5)	142 (21.4)	0.190
DBP, mmHg	80.0 (10.7)	79.8 (10.7)	81.5 (10.5)	**0.021**
BMI, kg/m^2^	24.5 (3.52)	24.3 (3.48)	25.9 (3.55)	**< 0.001**
WC, cm	83.6 (10.5)	83.0 (10.4)	88.5 (10.0)	**< 0.001**
TCH, mmol/L	4.68 (0.97)	4.66 (0.95)	4.82 (1.07)	**0.028**
LDL-C, mmol/L	3.24 (0.96)	3.23 (0.95)	3.37 (1.02)	**0.037**
HDL-C, mmol/L	1.28 (0.34)	1.30 (0.34)	1.16 (0.31)	**< 0.001**
Total triglycerides, mmol/L	1.36 (1.03)	1.30 (0.94)	1.81 (1.52)	**< 0.001**
Glu, mmol/L	5.78 (1.61)	5.79 (1.66)	5.65 (1.02)	0.062
Insulin, μU/mL	15.2 (8.88)	14.8 (7.80)	18.2 (15.0)	**< 0.001**
HbA1c, %	5.96 (1.05)	5.96 (1.08)	5.96 (0.74)	0.960
HOMA-IR	1.79 (1.12)	1.77 (1.11)	2.02 (1.32)	**< 0.001**
ALT, U/L	21.4 (19.5)	20.9 (20.0)	25.4 (13.0)	**< 0.001**
AST, U/L	30.7 (15.1)	30.1 (15.4)	35.4 (11.4)	**< 0.001**
GGT, U/L	32.3 (35.4)	31.1 (35.9)	43.1 (28.0)	**< 0.001**
Creatinine, mg/dL	56.5 (15.4)	54.9 (12.8)	71.0 (25.4)	**< 0.001**
Urea nitrogen, mmol/L	5.67 (1.59)	5.59 (1.47)	6.38 (2.30)	**< 0.001**
Uric acid, μmol/L	286 (82.2)	268 (64.1)	439 (57.4)	**< 0.001**
eGFR, mL/min/1.73 m^2^	86.5 (13.9)	88.0 (13.2)	73.5 (13.2)	**< 0.001**

Data are presented as mean (standard deviation) or median (interquartile range) for continuous variables, and count (percentage) for categorical variables. Percentages may not add up to 100% because of rounding. Characteristics were compared between participants with and without hyperuricemia using Student’s *t*-test or Wilcoxon’s Signed Rank test for continuous variables, and chi-squared test for categorical variables. Statistically significant *p*-values (< 0.05) are indicated in boldface

*ALT* alanine aminotransferase, *AST* aspartate transaminase, *BMI* body mass index, *DBP* diastolic blood pressure, *eGFR* estimated glomerular filtration rate, *GGT* γ-glutamyl transpeptidase, *Glu* fasting glucose, *HbA1c* glycated hemoglobin, *HDL-C* high-density lipoprotein cholesterol, *HOMA-IR* homeostatic model assessment of insulin resistance, *LDL-C* low-density lipoprotein cholesterol, *SBP* systolic blood pressure, *TCH* total cholesterol, *WC* waist circumference

### Lipidomic Profiles and Uric Acid

**Fig. 1:**
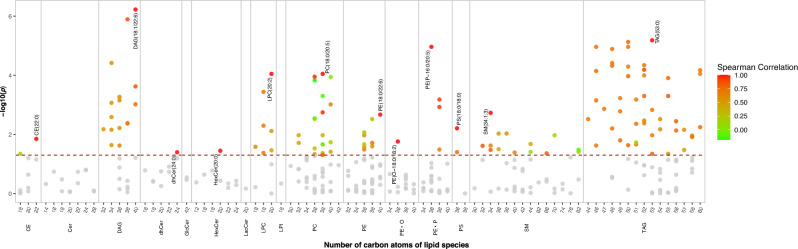
Manhattan plot for the associations of 350 lipids with uric acid within each subclass. *p-*values were derived from multivariable linear regression models after multiple corrections using the Benjamini–Hochberg method. Models were adjusted for demographic (sex, age, region, residence, education) and lifestyle (smoking status, drinking status, physical activity) information, metabolic traits (body mass index, γ-glutamyl transpeptidase, creatinine, homeostatic model assessment of insulin resistance, total triglycerides, and total cholesterol), disease (hypertension, family history of chronic diseases) and medication status (antihypertensive or lipid-lowering medicines). Colored circles denoted lipids that exhibited a significant association with uric acid, and the color gradient signified the Spearman correlation between the most significant uric acid-associated lipid species (selected based on the minimum *p*-values and labeled within their respective subclasses) and other lipid species within those subclasses. The dashed line indicates a cutoff of *p* < 0.05. *CE* cholesteryl ester, *Cer* ceramide, *DAG* diacylglycerol, *dhCer* dihydroceramide, *LPC* lysophosphatidylcholine, *LPI* lysophosphatidylinositol, *PC* phosphatidylcholine, *PE* phosphatidylethanolamine, *PS* phosphatidylserine, *SM* sphingomyelin, *SM (OH)* hydroxyl-sphingomyelin (with one additional hydroxyl), *SM (2OH)* hydroxyl-sphingomyelin (with two additional hydroxyls)

After multivariable adjustment, 123 lipids out of 350 lipids were significantly associated with UA (*p* < 0.05). Of them, 87 lipids [16 DAGs, 45 TAGs, 11 PCs, eight PEs, one PS, three SMs, two CEs, and one dihydroceramide (dhCer)] were positively associated with UA (*β* range: 3.35–10.38, *p* < 0.05); whereas 36 lipids (nine PCs, seven LPCs, six PEs, 11 SMs, one ceramide, and two TAGs) were inversely associated with UA (*β* range: − 6.38 to − 3.50, *p* < 0.05). Twelve lipids were identified as the top signature in each subclass, including [PC (18:0/20:5), LPC (20:2), PE (18:0/22:6), PE (P-16:0/20:5), PE (O-18:0/18:2), PS (18:0/18:0), hexosylceramides (20:0), dhCer (24:0), SM (34:1;3), CE (22:0), DAG (18:1/22:6), and TAG (53:0)] (Fig. 1).

### Lipidomic Profiles and Hyperuricemia

Concentrations of 186 out of 350 lipids differed significantly between participants with normal UA concentrations and those with HUA (*p* < 0.05). Out of these, the concentrations of 174 lipids were higher in the HUA group, including 77 GLs (25 DAGs, 52 TAGs), 71 GPs (33 PCs, 36 PEs, two PSs), 22 SPs (12 ceramides, 10 SMs), and four CEs. On the other hand, concentrations of 12 lipids including four PCs, four LPCs, and four SMs were lower in the HUA group (Table S2).

After multivariable adjustment, seven lipids were significantly associated with HUA; six of them [DAG (16:0/22:5), DAG (16:0/22:6), DAG (18:1/20:5), DAG (18:1/22:6), TAG (53:0), and PC (16:0/20:5)] had positive associations, while LPC (20:2) showed an inverse association (*p* < 0.05) (Table 2). There were significant modest correlations between these lipids [except for LPC (20:2)] and total triglycerides (0.50 < |*r*_s_| < 0.70), whereas weak but significant correlations with the anthropometric measures (WC, BMI) and other cardiometabolic traits (TCH, LDL-C, HDL-C, SBP, DBP, glucose, glycated hemoglobin (HbA1c), insulin, HOMA-IR, ALT, and GGT) were demonstrated (|*r*_s_| ≤ 0.40) (Fig. S3). In addition, they were significantly correlated with FA in the DNL pathway, especially 16:1n-7 (*r*_s_ = 0.32–0.41, *p* < 0.001) (Fig. S4).

**Table 2:** Risk ratios for lipids significantly associated with hyperuricemia

	Model 1			Model 2		
	RR (95% CI)	*p*-value	FDR	RR (95% CI)	*p*-value	FDR
DAG (16:0/22:5)	1.46 (1.29–1.64)	< 0.001	< 0.001	1.40 (1.18–1.66)	< 0.001	0.02
DAG (18:1/20:5)	1.36 (1.20–1.53)	< 0.001	< 0.001	1.31 (1.12–1.54)	< 0.001	0.03
DAG (16:0/22:6)	1.53 (1.35–1.73)	< 0.001	< 0.001	1.40 (1.18–1.65)	< 0.001	0.02
DAG (18:1/22:6)	1.52 (1.33–1.73)	< 0.001	< 0.001	1.35 (1.15–1.58)	< 0.001	0.02
TAG (53:0)	1.47 (1.29–1.67)	< 0.001	< 0.001	1.37 (1.15–1.63)	< 0.001	0.02
PC (16:0/20:5)	1.29 (1.12–1.48)	< 0.001	< 0.001	1.32 (1.14–1.53)	< 0.001	0.02
LPC (20:2)	0.78 (0.68–0.91)	< 0.001	0.002	0.76 (0.65–0.89)	< 0.001	0.03

*p-*values were identified by multivariable log-Poisson regression models. FDR values were corrected for multiple testing using the Benjamini–Hochberg method. Model 1: adjusted for age, sex, region, residence, educational attainment, physical activity, smoking status, drinking status, and family history of chronic diseases; Model 2: further adjusted for body mass index, γ-glutamyl transpeptidase, creatinine, homeostatic model assessment of insulin resistance, hypertension, medication status (antihypertensive or lipid-lowering medicines), total triglycerides, and total cholesterol

*DAG* diacylglycerol, *FDR* false-discovery rate, *LPC* lysophosphatidylcholine, *PC* phosphatidylcholine, *RR* risk ratio, *TAG* triacylglycerol

In the sensitivity analyses, the significant associations between the aforementioned lipids and HUA were not altered [RR: 0.79–1.53; all *p* < 0.05 except for LPC (20:2), which was marginally significant (*p* = 0.07)], after excluding participants taking lipid-lowering medications and those with declined kidney functions (Table S3). No significant interactions were detected in stratified analyses (all *p-*values for interaction > 0.05, Table S4). In the mediation analysis, RBP4 partially mediated the lipid-HUA associations after multiple adjustments (mediation proportion: 5–14%; *p* < 0.05) (Table S5).

### Network Analysis

**Fig. 2:**
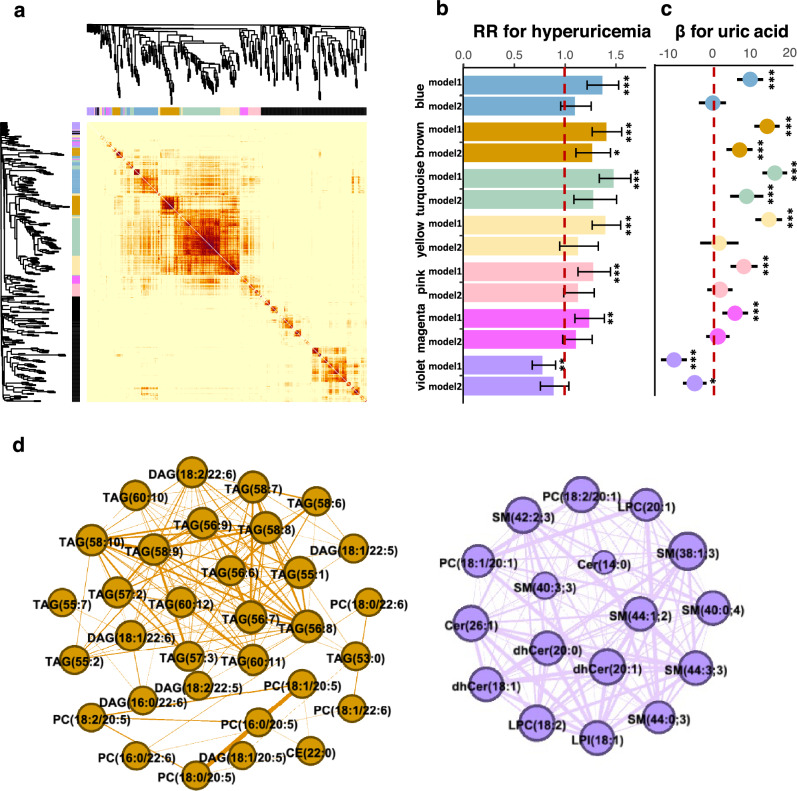
Weighted correlation network analysis of lipid profiles. **a** Cluster dendrogram and heatmap correlation of 350 lipids. The network analysis identified seven uric acid or hyperuricemia-related modules indicated by different colors (blue, brown, turquoise, yellow, pink, violet, and magenta). Modules that were not associated with uric acid or hyperuricemia were labeled grey. The information on each module was shown in Table S6. Intensity of red coloring indicates the strength of the correlation between pairs of lipids on a linear scale. **b** Relative risk (RR) for hyperuricemia per module score. Horizontal dashed line indicates RR = 1. Model 1: adjusted for age, sex, region, and residence; Model 2: further adjusted for educational attainment, physical activity, smoking status, drinking status, body mass index, γ-glutamyl transpeptidase, homeostatic model assessment of insulin resistance, creatinine, total triglycerides, total cholesterol, hypertension, and medication status (antihypertensive or lipid-lowering medicines). **c** Effect size (*β*) for uric acid per module score. **d** Two sub-networks (brown and violet) are represented by node and edge graph. Lipids are presented as node, and connection strength is represented by edge width (edges < 0.01 omitted)

Seven lipid modules were identified as significantly associated with UA or HUA after adjusting for age, sex, region, and residence among 15 modules clustered through WGCNA analysis (Fig. 2, Table S6), which were brown, turquoise, yellow, blue, pink, magenta, and violet modules. The correlations among all WGCNA modules are presented in Fig. S5. The brown and turquoise modules were characterized by TAGs/PCs/DAGs, of which about half were TAGs and most other lipids (e.g., DAGs, PCs, PEs,) contained FA acyl chain [saturated FA (SFAs, C16:0, C18:0) or monounsaturated FA (MUFA, C18:1)] in *sn-1* position and C20:5, C22:5 or C:22:6 in *sn*-2 position (Table S6 and Fig. 2d). The yellow module was mainly composed of TAGs/DAGs with more than half to be TAGs and DAGs containing SFAs/MUFAs/polyunsaturated FAs in *sn*-1 position, while the blue module was composed mainly of PEs containing FAs (C14:0, C16:0, and C18:0)/MUFA (C18:1) in *sn-1* position. The magenta and pink modules were characterized by PCs/LPCs/SMs, where more than half were PCs containing DNL fatty acyl chains. The violet module consisted of mainly SPs, LPCs, and PCs (Fig. 2d). The details of the WGCNA modules are presented in Table S6.

The violet module was the only one identified as inversely associated with UA and HUA. Per standard deviation increment of eigenlipid of the violet module, UA was decreased by 3.76 (95% CI 0.86–6.65; *p* < 0.001) μmol/L when fully adjusted, and RR of HUA was decreased by 0.78 (95% CI 0.68–0.91; *p* < 0.05) with age, sex, region, and residence adjusted, although the association became insignificant when further adjusting for lifestyle, metabolic traits, and medication use (Fig. 2b, c, Table S6). On the other hand, per standard deviation increment of eigenlipid of the brown module, UA was increased by 7.24 (95% CI 4.03–10.44; *p* < 0.001) μmol/L, and RR of HUA was increased by 1.27 (95% CI 1.11–1.45; *p* < 0.05) with multiple adjustments (Fig. 2b, c). Lipids inside the brown module with higher weights exhibited stronger correlations with HUA, further suggesting the brown module was a UA/HUA-related lipidomic module (Fig. S6).

### Dietary Factors and Individual or Collective UA/HUA-Related Lipids

**Fig. 3:**
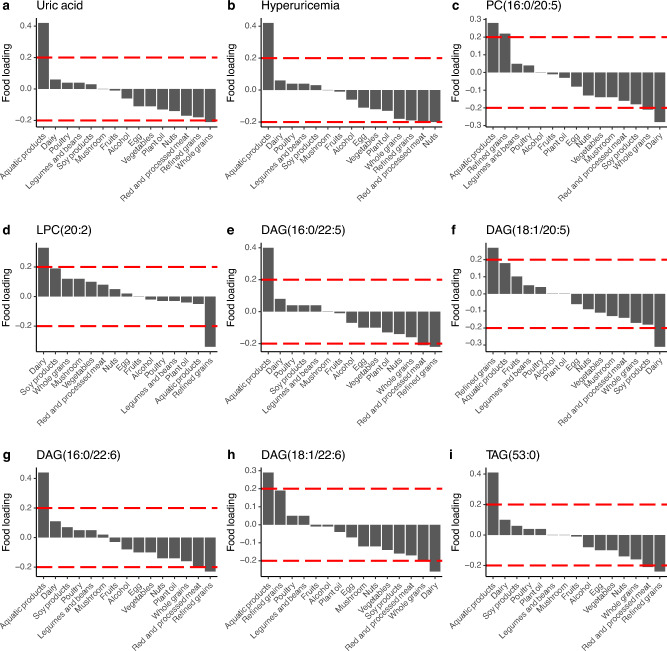
Food loadings were derived from the reduced rank regression for **a** uric acid, **b** hyperuricemia, **c** PC (16:0/20:5), **d** LPC (20:2), **e** DAG (16:0/22:5), **f** DAG (18:1/20:5), **g** DAG (16:0/22:6), **h** DAG (18:1/22:6), and **i** TAG (53:0). Model was adjusted for age, sex, region, residence. Food groups with absolute factor loading > 0.2 (dashed lines) were considered components of a dietary pattern representative of given lipids that were significantly associated with hyperuricemia. Each dietary traits pattern that explained the largest variance in each lipid metabolite was identified. *DAG* diacylglycerol, *LPC* lysophosphatidylcholine, *PC* phosphatidylcholine, *TAG* triacylglycerol

For seven lipids that were significantly associated with HUA risks, PC (16:0/20:5) and DAG (18:1/22:6) were both associated with high intakes of aquatic products but low consumption of dairy products; DAG (16:0/22:5), DAG (16:0/22:6), and TAG (53:0) were all associated with high intakes of aquatic products and low intakes of refined grains (Fig. 3). On the contrary, LPC (20:2) was related to high consumption of dairy products and low intakes of refined grains (Fig. 3). In addition, HUA risk could be explained by high intakes of aquatic products, and higher UA concentrations were associated with higher intakes of aquatic products and lower intakes of whole grains, representing a partially shared dietary pattern with those significant lipids.

## Discussion

Our study is the first population-based study that extensively examined the link between plasma UA, HUA, and lipidomic profiles. We found 123 lipid species that were significantly associated with UA after multivariable adjustment, predominantly GLs and GPs containing DNL fatty acyl chains. Of them, four DAGs, one PC, and one TAG (53:0) were positively associated with HUA risk, and associations might be partially mediated by RBP4. On the other hand, LPC (20:2) was inversely associated with HUA risk. Increased intakes of aquatic products were correlated with a higher risk of HUA, while high dairy consumption was correlated with low levels of HUA-related lipids.

Of all the UA/HUA-associated lipid metabolites, GLs were the most abundant ones as DAGs and TAGs, somewhat aligning with the finding of TAG as an independent risk factor for HUA in previous studies (Hou et al. 2019; Xu et al. 2022). To date, only one previous population-based case–control study has examined associations between lipidomic profiles and UA/HUA, and it reported 245 differential lipids spanning TAGs, DAGs, PCs, and PEs between 157 HUA patients and 88 healthy controls (Liu et al. 2022). In our study, not only five GLs, including DAG (16:0/22:5), DAG (16:0/22:6), DAG (18:1/20:5), DAG (18:1/22:6), TAG (53:0), were individually, but also the module containing TAGs/PCs/DAGs was collectively associated with higher HUA risk. As second messengers, intracellular DAGs are biologically active lipids and have been reported to induce insulin resistance (Erion and Shulman 2010), while TAGs could exert regulatory roles in FA oxidation and lipid synthesis (Coleman and Mashek 2011). Together, the findings from our study implicated a potential role for disturbed GL metabolism in the pathogenesis of HUA.

Glycerophospholipids (GPs), as the major lipids of cellular membranes, were another lipid subclass associated with UA and/or with HUA risk in our study. Previously, we documented positive associations of certain GPs (PCs 16:0/18:1, 18:0/16:1, 18:1/20:3, 16:0/16:1, 16:0/20:3 and 18:0/20:3; LPC 20:3; PE 16:0/16:1) with incident type 2 diabetes in the same population (Chen et al. 2022) and perturbed PCs could also attribute to insulin resistance, glucose intolerance (Jacobs et al. 2010; Raubenheimer et al. 2006), endoplasmic reticulum stress (Fu et al. 2011) and obesity (Jacobs et al. 2010), some of these abnormalities might also serve as common soil for pathogenesis for HUA. Although little was known about the human population, studies in rat models indicated that similar aberrations in GP metabolism could result in HUA (Yang et al. 2019). These alternated lipidomic signatures could be ascribed to inflammation-provoked aggregation of GPs stemming from the state of HUA (Yang et al. 2019). Although it remains unclear why LPC (20:2) is linked with the opposite risk of HUA, according to our study, Liu et al. revealed that LPCs, including LPC (20:2), were significantly suppressed under HUA, along with declined arachidonic acid and docosahexaenoic acid in HUA mouse models, which are preferred substrates of LPC acyltransferase 3 (LPCAT3) (Liu et al. 2020). These reductions in LPCs and LPCAT3 substrates might imply greater consumption driven by upregulated LPCAT3 under the condition of HUA. Nonetheless, future studies are necessary to elucidate the underlying mechanism of GPs in the pathogenesis of HUA.

When taking dietary factors into consideration, we found that high dairy consumption was tied to HUA-lower lipid LPC (20:2), whereas low dairy intakes were associated with HUA-prone lipids like PC (16:0/20:5) and DAG (18:1/22:6), though no significant associations were detected with UA/HUA risk per se. However, prior evidence has shown an inverse relationship between dairy intake and HUA risk. For example, NHANES-III results showed inverse dairy-UA associations (Choi et al. 2005). The observed discrepancies may stem from the fact of low dairy consumption with a median intake of 0.89 (interquartile range: 0.19–1.03) serving/day in this study population (Zong et al. 2014), far below intakes in Western populations (Choi et al. 2005). Indeed, no relationship was observed in African Americans who consumed less dairy, despite an inverse association between dairy and UA was observed in the white population (Beydoun et al. 2018). Another possible interpretation could be due to the fact that our study population consumed mainly whole-fat dairy products while the inverse associations for UA/HUA risk were primarily observed with low-fat dairy in western populations (Choi et al. 2004; Dalbeth et al. 2010) and the protective effect may be driven by dairy proteins (Choi et al. 2004; Gao et al. 2008) rather than lipids. Thus, the findings of a positive association between dairy proteins and LPCs (Hellmuth et al. 2018) may help explain the positive relationship between dairy consumption and LPC (20:2), a lipid tied to lower HUA risk.

Noteworthily, our study also highlighted that high HUA-related GLs and GPs were significantly correlated with FAs in the DNL pathway including myristic acid (14:0), palmitic acid (16:0), palmitoleic acid (16:1n-7), hexadecenoic acid (16:1n-9), and oleic acid (18:1n-9). A high-carbohydrate and low-fat diet could upregulate FAs in the hepatic DNL pathway (Schwarz et al. 2003). The high carbohydrate-to-fat ratio of the habitual diet (60.8%:27.0%) in this population (Zong et al. 2013) was found to be significantly associated with insulin resistance and 6-year incident type 2 diabetes, while in a 12-week full feeding intervention, we found that low-carbohydrate significantly reduced levels of DNL FAs like 14:0 and 16:1n-7 (Ma et al. 2021). Though underlying mechanistic links were not well-known between lipidomic signature and HUA risk, we noticed that the associations were partially mediated by RBP4, an adipokine secreted by adipocytes and hepatocytes. Studies from our and others showed that high circulating RBP4 was linked to dyslipidemia, fatty liver, cardiovascular diseases (Graham et al. 2006), as well as insulin resistance (Qi et al. 2007; Sun et al. 2014; Xu et al. 2009) via inflammation in adipose tissue (Moraes-Vieira et al. 2014, 2020) or by inducing expression of gluconeogenic enzyme such as phosphoenolpyruvate carboxykinase and by suppressing muscle insulin signalings (Yang et al. 2005). High RBP4 concentration is also independently associated with increased microalbuminuria risk, an early renal dysfunction indicator (Xu et al. 2009). Taken together, these adverse metabolic conditions associated with high fatty acyl chains in the hepatic DNL pathway or RBP4 may raise HUA risk by disrupting normal purine metabolism (Toledo-Ibelles et al. 2021) and/or impairing the kidneys' ability to adequately excrete UA (Muscelli et al. 1996).

The current study has several notable strengths. It is the largest population-based study that investigated the associations between lipidomic biomarkers and UA/HUA risk. The high-coverage targeted lipidomic approach enabled 350 lipids spanning multiple subclasses. Rigorous adjustment for an array of demographic, lifestyle, and cardiometabolic covariates was performed to minimize confounders. Sophisticated statistical techniques including network analysis allowed for an expanded systemic perspective and integration of lipidomic data with dietary patterns. Our study also has some limitations worth acknowledging. First, the cross-sectional nature restricts causal inference. Further longitudinal and mechanistic studies are needed to elucidate whether HUA is secondary to lipid disturbances or vice versa. Second, our population was limited to middle-aged and elderly Chinese, reducing the generalizability to other age groups or ethnicities. Finally, we did not have an independent cohort to replicate our findings, though the associations were robust across subgroups from two separate regions in Beijing and Shanghai.

## Conclusion

In summary, as the largest population-based lipidomic study of HUA to date, we found that predominantly GLs and GPs containing DNL fatty acyl chains were related to high UA/HUA risk while LPC (20:2) related to a lower risk. The lipidomic signatures may reflect HUA-related aquatic food consumption and may partially mediated by RBP4. Our findings offer new insights into HUA etiology and future studies are needed to elucidate whether HUA arises due to lipid abnormalities or the reverse.

## Supplementary Information

Below is the link to the electronic supplementary material.

Supplementary file 1 (DOCX 2691 KB)

## Data Availability

The datasets used during the current study are not publicly available due to ethics restrictions but are available from the corresponding author on reasonable request.

## Abbreviations

ALT: Alanine aminotransferase
AST: Aspartate transaminase
BMI: Body mass index
CE: Cholesteryl ester
CI: Confidence interval
DAG: Diacylglycerol
DBP: Diastolic blood pressure
DNL: De novo lipogenesis
dhCer: Dihydroceramide
eGFR: Estimated glomerular filtration rate
ELISA: Enzyme-linked immunosorbent assay
FA: Fatty acid
FDR: False-discovery rate
GGT: γ-Glutamyl transpeptidase
GL: Glycerolipid
GP: Glycerophospholipid
HbA1c: Glycated hemoglobin
HDL-C: High-density lipoprotein cholesterol
HOMA-IR: Homeostatic model assessment of insulin resistance
HUA: Hyperuricemia
LDL-C: Low-density lipoprotein cholesterol
LPC: Lysophosphatidylcholine
LPCAT3: Lysophosphatidylcholine acyltransferase 3
MUFA: Monounsaturated fatty acid
NHAPC: Nutrition and Health of Aging Population in China
PC: Phosphatidylcholine
PE: Phosphatidylethanolamine
PS: Phosphatidylserine
RBP4: Retinol binding protein 4
RR: Risk ratio
RRR: Reduced rank regression
*r*_s_: Spearman correlation coefficient
SBP: Systolic blood pressure
SFA: Saturated fatty acid
SM: Sphingomyelin
TAG: Triacylglycerol
TCH: Total cholesterol
UA: Uric acid
WC: Waist circumference
WGCNA: Weighted gene co-expression network analysis
