# Supplementary material for: Associations of Plasma Lipidomic Profiles with Uric Acid and Hyperuricemia Risk in Middle-Aged and Elderly Chinese
Source: Phenomics. 2024 Jul 30;4(4):352–64. doi: 10.1007/s43657-024-00157-x (PMC11584823; doi:10.1007/s43657-024-00157-x)
Supplement: Supplementary file 1 — (DOCX 2691 KB) [file 43657_2024_157_MOESM1_ESM.docx]

# Associations of Plasma Lipidomic Profiles with Uric Acid and Hyperuricemia Risk in Middle-Aged and Elderly Chinese

***Phenomics***

**Authors:**

Wanhui Kang, PhD, Xinming Xu, MBBS, Xiaowei Yang, PhD, Qingqing Wu, PhD, Shuning Li, BS, Keran Gao, MD, MPH, Rong Zeng, PhD, Liang Sun, PhD, Xu Lin, PhD

**Corresponding authors**:

Xu Lin, MD, PhD

Professor, Principal Investigator

Shanghai Institute of Nutrition and Health, Chinese Academy of Sciences, 320 Yue-yang Rd., Shanghai 200031, China

Key Laboratory of Systems Health Science of Zhejiang Province, School of Life Science, Hangzhou Institute for Advanced Study, University of Chinese Academy of Sciences, Hangzhou 310024, China

Phone: +86 21 54920249

Fax: +86 21 54920249

Email: xlin@sibs.ac.cn.

Liang Sun, MD, PhD

Professor, Principal Investigator

Department of Nutrition and Food Hygiene, School of Public Health, Institute of Nutrition, Fudan University, 130 Dongan Rd., Shanghai 200032, China

Shanghai Institute of Nutrition and Health, Chinese Academy of Sciences, 320 Yue-yang Rd., Shanghai 200031, China

Phone: +86-21-54237087

Fax: +86-21-54237087

Email: [sun_liang@fudan.edu.cn](mailto:sun_liang@fudan.edu.cn)

Rong Zeng, PhD

Professor, Principal Investigator

Shanghai Institute of Biochemistry and Cell Biology, Center for Excellence in Molecular Cell Science

Chinese Academy of Sciences, 320 Yue-yang Rd., Shanghai 200031, China

Key Laboratory of Systems Health Science of Zhejiang Province, School of Life Science, Hangzhou Institute for Advanced Study, University of Chinese Academy of Sciences, Hangzhou 310024, China

Phone: +86-21-54920160

Fax: +86-21-54921011

Email: [zr@sibs.ac.cn](mailto:zr@sibs.ac.cn)

# Table S1 Food groups and intake between those with and without hyperuricemia

| **Food Group** | **Examples Included** | **Total (n=2247)** | **Hyperuricemia** | | ***p*** |
| --- | --- | --- | --- | --- | --- |
|  |  |  | **No (n=2013)** | **Yes (n=234)** |  |
| Refined grain | Rice, Wheat and their products | 301 (232, 451) | 303 (235, 452) | 300(205, 400) | **0.001** |
| Whole grain | Glutinous rice, Millet, Corn, Sweet potatoes, etc. | 6.56 (1.54, 21.43) | 6.56 (1.64, 21.43) | 5.32 (1.37, 14.3) | 0.35 |
| Red and processed meat | Pork, Beef, Lamp, Donkey meat, Bacon, Ham, Sausage, Organ, Animal blood, etc. | 30.0 (13.2, 59.9) | 30.04 (13.1, 59.7) | 29.3 (14.1, 66.7) | 0.55 |
| Poultry | Chicken, Duck, Goose | 4.10 (1.37, 13.1) | 3.57 (1.23, 13.1) | 4.92 (1.64, 14.3) | 0.12 |
| Egg | Chicken egg, Duck egg, Quail egg, Salted egg, etc. | 38.2 (20.7, 65.4) | 38.21 (20.7, 65.4) | 38.2 (20.1, 65.7) | 0.94 |
| Dairy | Milk, Milk powder, Milk flake, Yogurt, Cheese, Ice-cream | 16.4 (0.00, 250) | 13.4 (0.00, 250) | 37.1 (0.00, 250) | 0.053 |
| Soy products | Tofu, Tofu curd, Bean noodle, etc. | 35.4 (14.3, 85.7) | 33.6 (14.3, 85.7) | 42.86 (14.8, 115) | **0.003** |
| Legumes and beans | Hyacinth bean, Jackbean, Lima-bean, Pea, Broad bean, Bean sprout, Mung bean, Red bean, Dry soy beans, etc. | 42.9 (22.2, 64.3) | 42.9 (22.1, 64.3) | 43.5 (24.2, 63.9) | 0.46 |
| Aquatic products | Freshwater fish, Saltwater fish, Shrimp, Crab, Shellfish (eg. Clam, Snail, Oysters), Soft fish (eg. Jellyfish, Cuttlelfish, Squid, Sea cucumber), Dried shrimp, Salted fish, Kelp, Laver, etc. | 26.1 (9.55, 55.9) | 24.6 (8.95, 53.7) | 39.7 (16.0, 72.3) | **<0.001** |
| Vegetables | Leafy vegetables, Root vegetables, Solanaceous vegetables, Stem vegetables, Gourd vegetables, etc. | 300 (191, 440) | 300 (189, 436) | 300 (199, 449) | 0.70 |
| Mushroom | Fresh mushroom, Straw mushroom, Needle mushroom, Shaggy mushroom, Oyster mushroom, Dry mushroom, etc. | 4.71 (0.82, 14.3) | 4.10 (0.82, 14.3) | 7.14 (1.64, 15.3) | **0.005** |
| Fruits | Apple, Orange, Bananas, Pear, Watermelon, Casaba, Pineapple, Strawberry, Longan, Sugarcane, Mango, Kiwi, Persimmon, Peach, etc. | 137 (61.5, 257) | 136 (60.2, 257) | 148 (70.3, 255) | 0.34 |
| Nuts | Peanut, Flavored sunflower seed, Walnut, Chestnut, etc. | 2.74 (0.27, 14.3) | 2.74 (0.26, 14.3) | 2.12 (0.27, 14.3) | 0.66 |
| Alcohol | Liquor, Beer, Wine, Rice wine | 0.00 (0.00, 9.02) | 0.00 (0.00, 8.20) | 0.00 (0.00, 13.6) | 0.60 |
| Vegetable oil | Soybean oil, Colza oil, Peanut oil, Sesame oil, Chili oil, etc. | 32.8 (24.6, 41.0) | 32.79 (24.6, 41.0) | 32.8 (24.6, 41.0) | 0.20 |

Data are presented as median (interquartile range) in units of g/day. Comparing baseline characteristics between participants with and without hyperuricemia using Wilcoxon’s Signed Rank tests. Statistically significant *p-*values (< 0.05) are indicated in boldface type

# Table S2 Plasma lipidomic profiles between those with and without hyperuricemia

| **Lipids(mg/L)** | **Hyperuricemia** | | ***p*** | **FDR** |
| --- | --- | --- | --- | --- |
|  | **No (n=2013)** | **Yes (n=234)** |  |  |
| PC (14:0/18:1) | 1.93 (0.80-4.67) | 2.10 (1.03-4.29) | **0.027** | **0.045** |
| PC (14:0/18:2) | 6.26 (2.89-13.6) | 6.96 (3.48-13.93) | **<0.001** | **<0.001** |
| PC (14:0/18:3) | 0.16 (0.05-0.50) | 0.17 (0.06-0.52) | **0.017** | **0.030** |
| PC (16:0/14:0) | 1.17 (0.36-3.77) | 1.33 (0.47-3.78) | **0.004** | **0.009** |
| PC (16:0/16:0) | 23.2 (14.8-36.2) | 24.2 (16.4-35.8) | **0.023** | **0.04** |
| PC (16:0/16:1) | 6.86 (2.08-22.6) | 7.85 (2.43-25.3) | **<0.001** | **<0.001** |
| PC (16:0/18:0) | 3.76 (2.25-6.29) | 4.13 (2.59-6.59) | **<0.001** | **<0.001** |
| PC (16:0/18:1) | 131 (74.7-230) | 138 (81.0-233) | **0.010** | **0.02** |
| PC (16:0/18:2) | 184 (128-264.) | 190 (132-274) | **0.014** | **0.027** |
| PC (16:0/18:3) | 3.96 (1.63-9.58) | 4.28 (1.69-10.87) | **<0.001** | **<0.001** |
| PC (16:0/20:1) | 0.56 (0.23-1.35) | 0.58 (0.27-1.26) | 0.97 | 0.98 |
| PC (16:0/20:2) | 2.88 (1.63-5.11) | 3.06 (1.76-5.34) | **<0.001** | **0.001** |
| PC (16:0/20:3) | 44.86 (21.3-94.7) | 49.5 (24.3-101) | **<0.001** | **<0.001** |
| PC (16:0/20:4) | 96.1 (53.9-171.2) | 95.6 (54.7-167) | 0.11 | 0.16 |
| PC (16:0/20:5) | 6.64 (2.13-20.67) | 7.83 (2.58-23.7) | **<0.001** | **0.001** |
| PC (16:0/22:4) | 3.76 (1.61-8.75) | 3.74 (1.67-8.36) | **0.015** | **0.027** |
| PC (16:0/22:5) | 13.8 (7.4-25.8) | 14.08 (8.02-24.7) | **0.028** | **0.047** |
| PC (16:0/22:6) | 30.4 (15.0-61.4) | 33.52 (17.5-64.2) | **0.011** | **0.02** |
| PC (16:1/18:1) | 3.78 (2.04-7.01) | 3.73 (2.08-6.67) | 0.83 | 0.87 |
| PC (16:1/18:2) | 1.60 (0.90-2.84) | 1.62 (0.96-2.74) | 0.18 | 0.25 |
| PC (18:0/16:1) | 0.26 (0.08-0.89) | 0.29 (0.08-1.03) | **<0.001** | **0.002** |
| PC (18:0/18:0) | 1.59 (0.88-2.86) | 1.75 (1.04-2.93) | **<0.001** | **<0.001** |
| PC (18:0/18:1) | 19.9 (10.82-36.7) | 21.27 (11.7-38.5) | **0.005** | **0.01** |
| PC (18:0/18:2) | 94.8 (57.2-157.3) | 99.8 (61.1-163) | **0.005** | **0.011** |
| PC (18:0/18:3) | 0.98 (0.34-2.87) | 1.07 (0.34-3.32) | **<0.001** | **<0.001** |
| PC (18:0/20:2) | 1.26 (0.60-2.64) | 1.38 (0.70-2.75) | **<0.001** | **<0.001** |
| PC (18:0/20:3) | 24.8 (11.4-53.9) | 27.1 (12.9-56.9) | **<0.001** | **<0.001** |
| PC (18:0/20:4) | 79.3 (47.3-133) | 77.5 (47.4-126) | 0.31 | 0.40 |
| PC (18:0/20:5) | 4.60 (1.51-14.3) | 5.35 (1.80-15.9) | **0.001** | **0.003** |
| PC (18:0/22:4) | 2.50 (1.10-5.45) | 2.47 (1.11-5.48) | **0.003** | **0.006** |
| PC (18:0/22:5) | 8.80 (4.30-18.0) | 9.22 (4.56-18.7) | **<0.001** | **<0.001** |
| PC (18:0/22:6) | 34.9 (18.2-66.8) | 39.5 (22.4-69.5) | **<0.001** | **<0.001** |
| PC (18:1/18:1) | 10.7 (5.70-20.1) | 10.2 (5.80-18.07) | **0.024** | **0.042** |
| PC (18:1/18:2) | 50.5 (30.5-83.6) | 50.5 (30.9-82.7) | 0.93 | 0.95 |
| PC (18:1/18:3) | 0.70 (0.29-1.67) | 0.70 (0.27-1.82) | **0.017** | **0.031** |
| PC (18:1/20:1) | 0.16 (0.05-0.49) | 0.15 (0.05-0.42) | **0.04** | 0.066 |
| PC (18:1/20:2) | 0.80 (0.41-1.56) | 0.83 (0.43-1.57) | **0.023** | **0.040** |
| PC (18:1/20:3) | 12.4 (5.82-26.27) | 13.0 (6.29-26.7) | **<0.001** | **<0.001** |
| PC (18:1/20:4) | 38.3 (21.6-67.9) | 36.1 (20.9-62.5) | 0.66 | 0.73 |
| PC (18:1/20:5) | 2.25 (0.79-6.45) | 2.50 (0.90-6.95) | **0.020** | **0.036** |
| PC (18:1/22:5) | 4.49 (2.34-8.60) | 4.47 (2.39-8.35) | **0.020** | **0.036** |
| PC (18:1/22:6) | 17.3 (9.64-31.0) | 18.5 (11.0-31.4) | **0.011** | **0.020** |
| PC (18:2/16:1) | 2.73 (1.41-5.29) | 2.75 (1.50-5.03) | 0.61 | 0.70 |
| PC (18:2/18:2) | 14.4 (6.84-30.2) | 14.1 (6.81-29.1) | 0.17 | 0.24 |
| PC (18:2/18:3) | 0.47 (0.19-1.15) | 0.46 (0.18-1.17) | 0.65 | 0.73 |
| PC (18:2/20:1) | 0.47 (0.17-1.26) | 0.43 (0.18-0.99) | **0.009** | **0.019** |
| PC (18:2/20:2) | 0.59 (0.30-1.16) | 0.56 (0.29-1.10) | 0.23 | 0.31 |
| PC (18:2/20:3) | 3.21 (1.53-6.72) | 3.28 (1.57-6.82) | **0.014** | **0.027** |
| PC (18:2/20:4) | 10.2 (5.64-18.3) | 9.54 (5.49-16.6) | 0.24 | 0.32 |
| PC (18:2/20:5) | 0.50 (0.18-1.39) | 0.55 (0.20-1.50) | 0.092 | 0.14 |
| PC (18:2/22:5) | 0.76 (0.41-1.43) | 0.70 (0.38-1.31) | 0.086 | 0.13 |
| PC (18:2/22:6) | 2.755 (1.444-5.254) | 2.75 (1.45-5.24) | 0.242 | 0.32 |
| PC (20:0/20:4) | 1.85 (0.92-3.72) | 1.80 (0.90-3.59) | 0.205 | 0.279 |
| PC (20:0/22:4) | 0.97 (0.38-2.45) | 0.98 (0.39-2.49) | **<0.001** | **0.002** |
| LPC (16:0) | 18.7 (12.7-27.5) | 18.9 (13.0-27.4) | 0.343 | 0.433 |
| LPC (16:1) | 0.83 (0.41-1.68) | 0.83 (0.43-1.62) | 0.311 | 0.400 |
| LPC (18:0) | 23.6 (15.1-37.0) | 23.5 (16.0-35.5) | 0.911 | 0.94 |
| LPC (18:1) | 15.1 (8.51-26.8) | 14.1 (8.16-24.4) | **0.004** | **0.008** |
| LPC (18:2) | 21.7 (12.5-38.0) | 20.6 (12.5-33.9) | **<0.001** | **0.001** |
| LPC (20:0) | 1.31 (0.93-1.83) | 1.27 (0.91-1.76) | 0.061 | 0.096 |
| LPC (20:1) | 0.46 (0.18-1.15) | 0.41 (0.17-0.96) | **0.005** | **0.011** |
| LPC (20:2) | 0.44 (0.23-0.82) | 0.41 (0.22-0.74) | **0.001** | **0.003** |
| LPC (20:3) | 1.30 (0.63-2.69) | 1.27 (0.63-2.58) | 0.432 | 0.526 |
| LPC (20:4) | 1.71 (0.83-3.53) | 1.55 (0.77-3.10) | **0.042** | 0.070 |
| LPI (18:1) | 0.16 (0.07-0.33) | 0.15 (0.08-0.31) | 0.48 | 0.57 |
| PE (14:0/18:2) | 0.005 (0.001-0.020) | 0.006 (0.002-0.024) | **0.002** | **0.004** |
| PE (16:0/16:0) | 0.014 (0.005-0.037) | 0.016 (0.006-0.040) | **0.003** | **0.006** |
| PE (16:0/16:1) | 0.037 (0.007-0.210) | 0.048 (0.009-0.247) | **<0.001** | **0.002** |
| PE (16:0/18:1) | 0.347 (0.131-0.920) | 0.417 (0.166-1.051) | **<0.001** | **<0.001** |
| PE (16:0/18:2) | 1.22 (0.43-3.46) | 1.50 (0.54-4.15) | **<0.001** | **<0.001** |
| PE (16:0/18:3) | 0.038 (0.010-0.138) | 0.045 (0.013-0.16) | **<0.001** | **<0.001** |
| PE (16:0/20:1) | 0.008 (0.002-0.032) | 0.010 (0.003-0.033) | **0.032** | 0.053 |
| PE (16:0/20:2) | 0.025 (0.009-0.067) | 0.027 (0.010-0.076) | **0.013** | **0.025** |
| PE (16:0/20:3) | 0.15 (0.05-0.44) | 0.17 (0.06-0.50) | **<0.001** | **<0.001** |
| PE (16:0/20:4) | 0.99 (0.43-2.25) | 1.11 (0.52-2.37) | **0.001** | **0.003** |
| PE (16:0/20:5) | 0.027 (0.007-0.102) | 0.034 (0.01-0.12) | **<0.001** | **<0.001** |
| PE (16:0/22:4) | 0.104 (0.031-0.347) | 0.111 (0.034-0.367) | **0.030** | 0.051 |
| PE (16:0/22:5) | 0.30 (0.11-0.85) | 0.34 (0.14-0.86) | **0.001** | **0.003** |
| PE (16:0/22:6) | 0.85 (0.31-2.33) | 1.06 (0.41-2.75) | **<0.001** | **<0.001** |
| PE (18:0/14:0) | 0.028 (0.01-0.08) | 0.034 (0.01-0.09) | **<0.001** | **<0.001** |
| PE (18:0/16:0) | 0.013 (0.005-0.03) | 0.015 (0.006-0.038) | **<0.001** | **<0.001** |
| PE (18:0/16:1) | 0.018 (0.005-0.061) | 0.022 (0.007-0.071) | **<0.001** | **<0.001** |
| PE (18:0/18:0) | 0.15 (0.076-0.29) | 0.17 (0.082-0.33) | **0.002** | **0.004** |
| PE (18:0/18:1) | 0.54 (0.22-1.34) | 0.66 (0.27-1.63) | **<0.001** | **<0.001** |
| PE (18:0/18:2) | 2.78 (1.03-7.53) | 3.46 (1.24-9.63) | **<0.001** | **<0.001** |
| PE (18:0/18:3) | 0.05 (0.01-0.16) | 0.06 (0.02-0.19) | **<0.001** | **<0.001** |
| PE (18:0/20:2) | 0.02 (0.007-0.05) | 0.02 (0.008-0.6) | **<0.001** | **<0.001** |
| PE (18:0/20:3) | 0.47 (0.171-1.304) | 0.574 (0.212-1.558) | **<0.001** | **<0.001** |
| PE (18:0/20:4) | 3.60 (1.612-8.035) | 4.07 (1.79-9.24) | **<0.001** | **<0.001** |
| PE (18:0/20:5) | 0.09 (0.03-0.30) | 0.12 (0.04-0.34) | **<0.001** | **<0.001** |
| PE (18:0/22:4) | 0.15 (0.04-0.51) | 0.17 (0.05-0.56) | **0.003** | **0.006** |
| PE (18:0/22:5) | 0.30 (0.11-0.81) | 0.35 (0.13-0.93) | **<0.001** | **<0.001** |
| PE (18:0/22:6) | 0.68 (0.23-2.01) | 0.92 (0.34-2.47) | **<0.001** | **<0.001** |
| PE (18:1/16:1) | 0.043 (0.020-0.093) | 0.047 (0.021-0.103) | **0.047** | 0.077 |
| PE (18:1/18:1) | 0.52 (0.22-1.24) | 0.57 (0.24-1.33) | 0.27 | 0.36 |
| PE (18:1/18:2) | 1.42 (0.54-3.73) | 1.65 (0.61-4.44) | **<0.001** | **<0.001** |
| PE (18:1/18:3) | 0.031 (0.010-0.096) | 0.035 (0.012-0.103) | **0.002** | **0.004** |
| PE (18:1/20:1) | 0.011 (0.003-0.035) | 0.012 (0.004-0.034) | 0.692 | 0.752 |
| PE (18:1/20:2) | 0.020 (0.008-0.050) | 0.022 (0.009-0.055) | 0.053 | 0.085 |
| PE (18:1/20:3) | 0.230 (0.086-0.619) | 0.266 (0.099-0.715) | **<0.001** | **<0.001** |
| PE (18:1/20:4) | 1.60 (0.70-3.67) | 1.74 (0.74-4.07) | **0.01** | **0.019** |
| PE (18:1/20:5) | 0.04 (0.01-0.14) | 0.05 (0.02-0.15) | **<0.001** | **0.001** |
| PE (18:1/22:4) | 0.026 (0.009-0.079) | 0.027 (0.008-0.086) | 0.73 | 0.79 |
| PE (18:1/22:5) | 0.15 (0.06-0.40) | 0.18 (0.07-0.45) | **<0.001** | **<0.001** |
| PE (18:1/22:6) | 0.38 (0.14-1.02) | 0.48 (0.18-1.22) | **<0.001** | **<0.001** |
| PE (18:2/16:1) | 0.114 (0.045-0.289) | 0.124 (0.049-0.315) | 0.75 | 0.80 |
| PE (18:2/18:2) | 0.147 (0.055-0.398) | 0.158 (0.057-0.439) | 0.63 | 0.71 |
| PE (18:2/18:3) | 0.010 (0.003-0.037) | 0.011 (0.003-0.040) | **0.049** | 0.080 |
| PE (18:2/20:1) | 0.013 (0.004-0.041) | 0.014 (0.005-0.042) | 0.34 | 0.43 |
| PE (18:2/20:2) | 0.013 (0.004-0.041) | 0.014 (0.004-0.044) | 0.75 | 0.80 |
| PE (18:2/20:3) | 0.046 (0.017-0.125) | 0.050 (0.018-0.142) | 0.053 | 0.085 |
| PE (18:2/20:4) | 0.30 (0.13-0.71) | 0.31 (0.13-0.75) | 0.85 | 0.89 |
| PE (18:2/22:6) | 0.26 (0.10-0.69) | 0.31 (0.12-0.81) | **<0.001** | **<0.001** |
| PE (O-16:0/18:1) | 0.056 (0.025-0.125) | 0.059 (0.027-0.127) | 0.54 | 0.62 |
| PE (O-16:0/18:2) | 0.127 (0.053-0.303) | 0.134 (0.057-0.316) | 0.48 | 0.57 |
| PE (O-16:0/20:4) | 0.356 (0.145-0.878) | 0.380 (0.163-0.887) | 0.43 | 0.53 |
| PE (O-16:0/22:4) | 0.069 (0.027-0.177) | 0.068 (0.026-0.179) | 0.91 | 0.94 |
| PE (O-16:0/22:5) | 0.18 (0.08-0.40) | 0.19 (0.09-0.41) | 0.12 | 0.17 |
| PE (O-16:0/22:6) | 0.20 (0.09-0.43) | 0.22 (0.10-0.47) | 0.12 | 0.17 |
| PE (O-18:0/18:1) | 0.101 (0.047-0.215) | 0.107 (0.052-0.219) | 0.15 | 0.21 |
| PE (O-18:0/18:2) | 0.181 (0.082-0.400) | 0.191 (0.087-0.417) | 0.17 | 0.24 |
| PE (O-18:0/20:3) | 0.036 (0.015-0.088) | 0.038 (0.016-0.091) | 0.276 | 0.360 |
| PE (O-18:0/20:4) | 0.51 (0.21-1.28) | 0.53 (0.22-1.26) | 0.778 | 0.820 |
| PE (O-18:0/20:5) | 0.04 (0.02-0.11) | 0.05 (0.02-0.12) | 0.100 | 0.149 |
| PE (O-18:0/22:4) | 0.21 (0.07-0.66) | 0.22 (0.07-0.71) | 0.411 | 0.505 |
| PE (O-18:0/22:5) | 0.117 (0.051-0.267) | 0.119 (0.051-0.278) | 0.348 | 0.437 |
| PE (O-18:0/22:6) | 0.19 (0.08-0.43) | 0.21 (0.09-0.46) | 0.206 | 0.280 |
| PE (P-16:0/18:1) | 0.35 (0.19-0.66) | 0.36 (0.19-0.69) | 0.699 | 0.757 |
| PE (P-16:0/18:2) | 0.99 (0.45-2.18) | 1.03 (0.47-2.23) | 0.941 | 0.954 |
| PE (P-16:0/20:2) | 0.01 (0.01-0.03) | 0.01 (0.01-0.02) | 0.546 | 0.628 |
| PE (P-16:0/20:3) | 0.25 (0.11-0.55) | 0.27 (0.13-0.56) | 0.283 | 0.368 |
| PE (P-16:0/20:4) | 2.57 (1.18-5.61) | 2.76 (1.28-5.93) | 0.239 | 0.318 |
| PE (P-16:0/20:5) | 0.07 (0.02-0.28) | 0.09 (0.02-0.34) | 0.097 | 0.146 |
| PE (P-16:0/22:4) | 0.40 (0.15-1.08) | 0.41 (0.14-1.17) | 0.685 | 0.746 |
| PE (P-16:0/22:5) | 0.72 (0.36-1.45) | 0.74 (0.36-1.53) | 0.118 | 0.172 |
| PE (P-16:0/22:6) | 0.93 (0.45-1.93) | 1.03 (0.50-2.16) | **0.014** | **0.026** |
| PE (P-18:0/16:1) | 0.024 (0.010-0.057) | 0.025 (0.009-0.065) | 0.094 | 0.142 |
| PE (P-18:0/18:1) | 0.295 (0.144-0.604) | 0.299 (0.148-0.601) | 0.925 | 0.949 |
| PE (P-18:0/18:2) | 1.47 (0.66-3.30) | 1.48 (0.67-3.28) | 0.498 | 0.585 |
| PE (P-18:0/20:3) | 0.42 (0.19-0.89) | 0.44 (0.21-0.92) | 0.328 | 0.419 |
| PE (P-18:0/20:4) | 4.90 (2.13-11.29) | 5.14 (2.21-11.96) | 0.453 | 0.545 |
| PE (P-18:0/20:5) | 0.21 (0.06-0.69) | 0.25 (0.08-0.82) | 0.062 | 0.097 |
| PE (P-18:0/22:4) | 0.43 (0.13-1.45) | 0.46 (0.12-1.67) | 0.449 | 0.541 |
| PE (P-18:0/22:5) | 0.66 (0.29-1.50) | 0.68 (0.29-1.60) | 0.463 | 0.555 |
| PE (P-18:0/22:6) | 1.29 (0.56-2.93) | 1.39 (0.59-3.28) | 0.345 | 0.434 |
| PE (P-18:1/16:0) | 0.06 (0.03-0.12) | 0.05 (0.03-0.11) | **0.049** | 0.079 |
| PE (P-18:1/18:1) | 0.37 (0.18-0.74) | 0.36 (0.19-0.70) | 0.219 | 0.296 |
| PE (P-18:1/18:2) | 1.062 (0.489-2.307) | 1.057 (0.496-2.255) | 0.339 | 0.431 |
| PE (P-18:1/20:3) | 0.30 (0.14-0.64) | 0.31 (0.15-0.64) | 0.73 | 0.786 |
| PE (P-18:1/20:4) | 3.73 (1.70-8.20) | 3.82 (1.78-8.22) | 0.877 | 0.911 |
| PE (P-18:1/20:5) | 0.13 (0.04-0.46) | 0.16 (0.04-0.54) | 0.114 | 0.168 |
| PE (P-18:1/22:4) | 0.11 (0.05-0.23) | 0.10 (0.05-0.24) | 0.377 | 0.468 |
| PE (P-18:1/22:5) | 0.190 (0.095-0.382) | 0.192 (0.094-0.391) | 0.363 | 0.452 |
| PE (P-18:1/22:6) | 1.08 (0.49-2.38) | 1.16 (0.52-2.59) | 0.508 | 0.594 |
| PE (P-18:2/18:2) | 0.061 (0.025-0.146) | 0.059 (0.024-0.148) | 0.409 | 0.503 |
| PE (P-18:2/20:4) | 0.75 (0.32-1.75) | 0.80 (0.35-1.80) | 0.243 | 0.321 |
| PE (P-18:2/22:6) | 0.27 (0.12-0.58) | 0.30 (0.14-0.64) | **0.012** | **0.023** |
| PS (18:0/18:0) | 0.32 (0.11-0.94) | 0.43 (0.16-1.14) | **<0.001** | **<0.001** |
| PS (18:0/18:1) | 0.14 (0.06-0.36) | 0.16 (0.06-0.40) | **0.008** | **0.015** |
| PS (18:2/20:4) | 0.21 (0.11-0.40) | 0.22 (0.11-0.42) | 0.677 | 0.741 |
| SM (2OH) C30:2 | 1.34 (0.64-2.79) | 1.46 (0.69-3.08) | **<0.001** | **<0.001** |
| SM (2OH) C32:1 | 2.31 (1.28-4.19) | 2.26 (1.26-4.05) | 0.936 | 0.952 |
| SM (2OH) C34:1 | 1.42 (0.79-2.57) | 1.40 (0.75-2.60) | 0.946 | 0.957 |
| SM (2OH) C40:0 | 20.78 (19.06-22.65) | 20.77 (18.97-22.74) | 0.416 | 0.509 |
| SM (2OH) C40:1 | 45.55 (39.82-52.10) | 45.46 (40.34-51.24) | 0.676 | 0.741 |
| SM (2OH) C42:4 | 5.07 (3.21-8.01) | 4.93 (3.15-7.72) | 0.994 | 0.994 |
| SM (OH) C32:3 | 3.56 (1.88-6.73) | 3.63 (1.87-7.05) | 0.179 | 0.247 |
| SM (OH) C34:0 | 16.30 (10.52-25.24) | 16.00 (10.21-25.08) | 0.955 | 0.963 |
| SM (OH) C34:1 | 41.13 (29.68-56.99) | 40.50 (29.63-55.36) | 0.885 | 0.917 |
| SM (OH) C34:2 | 1.89 (1.06-3.37) | 1.90 (1.02-3.55) | 0.766 | 0.813 |
| SM (OH) C36:1 | 14.88 (9.82-22.55) | 15.53 (9.98-24.18) | **<0.001** | **<0.001** |
| SM (OH) C36:2 | 8.89 (6.06-13.03) | 8.95 (6.02-13.31) | 0.720 | 0.777 |
| SM (OH) C36:3 | 55.29 (39.94-76.55) | 55.70 (41.06-75.57) | 0.087 | 0.132 |
| SM (OH) C38:1 | 4.36 (2.84-6.69) | 4.40 (3.04-6.37) | 0.979 | 0.982 |
| SM (OH) C38:2 | 11.72 (9.06-15.17) | 11.63 (8.97-15.07) | 0.169 | 0.237 |
| SM (OH) C38:3 | 24.60 (19.94-30.35) | 24.64 (20.02-30.31) | **0.034** | 0.057 |
| SM (OH) C40:1 | 3.62 (1.64-8.01) | 3.81 (1.64-8.83) | **<0.001** | **0.002** |
| SM (OH) C40:2 | 12.74 (6.62-24.51) | 13.14 (6.46-26.75) | **0.003** | **0.007** |
| SM (OH) C40:3 | 50.14 (43.00-58.48) | 50.08 (43.56-57.58) | 0.479 | 0.571 |
| SM (OH) C40:4 | 8.56 (1.83-40.14) | 9.05 (7.00-11.69) | 0.605 | 0.689 |
| SM (OH) C42:2 | 2.45 (1.45-4.13) | 2.29 (0.83-6.34) | **0.005** | **0.011** |
| SM (OH) C42:3 | 6.23 (4.22-9.18) | 5.87 (4.10-8.39) | **<0.001** | **0.001** |
| SM (OH) C42:4 | 0.58 (0.26-1.26) | 0.57 (0.24-1.37) | 0.287 | 0.371 |
| SM (OH) C44:0 | 1.06 (0.53-2.12) | 0.98 (0.53-1.79) | **0.026** | **0.044** |
| SM (OH) C44:1 | 1.21 (0.76-1.94) | 1.17 (0.73-1.86) | 0.381 | 0.471 |
| SM (OH) C44:3 | 1.85 (1.29-2.66) | 1.81 (1.26-2.60) | 0.083 | 0.128 |
| SM C32:1 | 5.20 (2.73-9.90) | 5.63 (2.91-10.92) | **<0.001** | **<0.001** |
| SM C34:0 | 13.28 (8.66-20.38) | 13.41 (8.70-20.67) | **0.008** | **0.016** |
| SM C34:1 | 2.92 (1.70-5.02) | 2.96 (1.72-5.08) | 0.148 | 0.212 |
| SM C34:2 | 14.61 (10.51-20.29) | 14.85 (10.78-20.45) | **0.011** | **0.020** |
| SM C36:0 | 8.13 (4.86-13.61) | 8.51 (4.91-14.75) | **<0.001** | **<0.001** |
| SM C36:1 | 9.85 (4.61-21.06) | 10.75 (5.30-21.82) | **<0.001** | **<0.001** |
| SM C36:2 | 7.09 (5.08-9.89) | 6.96 (5.16-9.39) | 0.358 | 0.448 |
| SM C38:0 | 9.52 (7.52-12.04) | 9.54 (7.45-12.21) | **0.047** | 0.077 |
| SM C38:1 | 46.34 (33.45-64.21) | 46.35 (34.19-62.82) | 0.648 | 0.724 |
| SM C40:0 | 50.70 (41.65-61.72) | 50.92 (42.47-61.06) | 0.057 | 0.090 |
| SM C40:1 | 45.67 (32.57-64.05) | 45.56 (33.46-62.02) | 0.616 | 0.698 |
| SM C42:0 | 16.77 (12.98-21.66) | 16.55 (12.93-21.18) | 0.676 | 0.741 |
| SM C42:1 | 37.83 (27.16-52.70) | 37.34 (26.72-52.20) | 0.834 | 0.873 |
| SM C42:2 | 46.51 (38.60-56.05) | 46.76 (39.53-55.31) | 0.052 | 0.084 |
| SM C42:3 | 43.24 (35.30-52.98) | 43.14 (35.73-52.07) | **0.015** | **0.027** |
| SM C44:1 | 3.41 (1.75-6.65) | 3.17 (0.97-10.31) | 0.171 | 0.238 |
| SM C44:3 | 6.83 (3.98-11.70) | 6.86 (3.94-11.95) | **0.002** | **0.004** |
| Cer (d18:0/16:0) | 0.022 (0.012-0.041) | 0.022 (0.012-0.042) | 0.553 | 0.635 |
| Cer (d18:0/18:0) | 0.016 (0.007-0.035) | 0.016 (0.007-0.036) | 0.522 | 0.609 |
| Cer (d18:0/18:1) | 0.011 (0.004-0.028) | 0.012 (0.005-0.028) | 0.193 | 0.264 |
| Cer (d18:0/20:0) | 0.009 (0.004-0.022) | 0.009 (0.004-0.024) | 0.100 | 0.149 |
| Cer (d18:0/20:1) | 0.007 (0.003-0.016) | 0.008 (0.004-0.018) | **0.002** | **0.004** |
| Cer (d18:0/22:0) | 0.049 (0.021-0.114) | 0.054 (0.023-0.127) | **0.005** | **0.010** |
| Cer (d18:0/22:1) | 0.115 (0.047-0.283) | 0.128 (0.054-0.300) | **<0.001** | **0.002** |
| Cer (d18:0/24:0) | 0.123 (0.052-0.291) | 0.134 (0.057-0.316) | **<0.001** | **<0.001** |
| Cer (d18:0/24:1) | 0.098 (0.051-0.187) | 0.105 (0.054-0.203) | **0.003** | **0.006** |
| Cer (d18:1/14:0) | 0.140 (0.104-0.188) | 0.139 (0.104-0.185) | 0.932 | 0.951 |
| Cer (d18:1/16:0) | 0.480 (0.309-0.746) | 0.485 (0.304-0.773) | 0.188 | 0.257 |
| Cer (d18:1/18:0) | 0.32 (0.17-0.61) | 0.33 (0.18-0.62) | 0.063 | 0.098 |
| Cer (d18:1/18:1) | 0.050 (0.023-0.108) | 0.055 (0.025-0.123) | **0.003** | **0.006** |
| Cer (d18:1/20:0) | 0.12 (0.06-0.23) | 0.13 (0.06-0.25) | **0.023** | **0.040** |
| Cer (d18:1/20:1) | 0.070 (0.037-0.133) | 0.073 (0.036-0.146) | 0.079 | 0.122 |
| Cer (d18:1/22:0) | 0.96 (0.47-1.97) | 1.03 (0.49-2.13) | **0.002** | **0.004** |
| Cer (d18:1/22:1) | 0.55 (0.26-1.15) | 0.58 (0.27-1.23) | **0.013** | **0.025** |
| Cer (d18:1/24:0) | 2.86 (1.27-6.48) | 3.05 (1.33-6.98) | **<0.001** | **<0.001** |
| Cer (d18:1/24:1) | 3.00 (1.74-5.17) | 3.14 (1.81-5.42) | **0.004** | **0.008** |
| Cer (d18:1/26:0) | 0.56 (0.26-1.24) | 0.58 (0.25-1.30) | 0.263 | 0.345 |
| Cer (d18:1/26:1) | 0.041 (0.019-0.087) | 0.042 (0.020-0.086) | 0.493 | 0.581 |
| GluCer (d18:0/24:0) | 0.064 (0.029-0.142) | 0.060 (0.025-0.145) | 0.772 | 0.816 |
| GluCer (d18:0/24:1) | 0.042 (0.019-0.091) | 0.039 (0.018-0.087) | 0.529 | 0.615 |
| HexCer (d18:1/12:0) | 0.036 (0.015-0.084) | 0.037 (0.016-0.085) | 0.161 | 0.227 |
| HexCer (d18:1/16:0) | 1.13 (0.55-2.31) | 1.05 (0.47-2.37) | 0.639 | 0.719 |
| HexCer (d18:1/18:0) | 0.61 (0.30-1.26) | 0.59 (0.27-1.29) | 0.646 | 0.724 |
| HexCer (d18:1/20:0) | 0.15 (0.07-0.30) | 0.14 (0.07-0.28) | 0.308 | 0.398 |
| HexCer (d18:1/20:1) | 0.101 (0.048-0.210) | 0.098 (0.043-0.222) | 0.594 | 0.679 |
| HexCer (d18:1/22:0) | 1.23 (0.58-2.61) | 1.18 (0.52-2.68) | 0.672 | 0.741 |
| HexCer (d18:1/22:1) | 0.67 (0.33-1.39) | 0.66 (0.30-1.43) | 0.652 | 0.725 |
| HexCer (d18:1/24:0) | 1.63 (0.75-3.56) | 1.57 (0.67-3.67) | 0.492 | 0.581 |
| HexCer (d18:1/24:1) | 2.15 (1.06-4.35) | 2.02 (0.91-4.52) | 0.856 | 0.891 |
| LacCer (d18:1/20:1) | 0.029 (0.014-0.057) | 0.030 (0.015-0.059) | **0.005** | **0.010** |
| CE (18:0) | 9.37 (3.46-25.37) | 10.33 (4.24-25.20) | **<0.001** | **0.001** |
| CE (18:1) | 339.67 (191.30-603.09) | 351.76 (208.48-593.53) | 0.228 | 0.307 |
| CE (18:2) | 814.64 (488.69-1358.00) | 851.88 (500.92-1448.72) | **0.022** | **0.038** |
| CE (18:3) | 76.74 (44.86-131.27) | 80.01 (45.01-142.23) | **0.024** | **0.042** |
| CE (20:2) | 3.40 (1.56-7.44) | 3.51 (1.67-7.36) | 0.059 | 0.093 |
| CE (20:3) | 3.51 (1.77-6.98) | 3.64 (1.84-7.20) | **0.011** | **0.021** |
| CE (20:4) | 68.15 (35.18-132.01) | 67.16 (35.33-127.69) | 0.544 | 0.628 |
| CE (20:5) | 58.77 (27.64-124.95) | 59.31 (28.70-122.57) | 0.116 | 0.17 |
| CE (22:0) | 2.48 (0.75-8.15) | 2.67 (0.86-8.28) | 0.448 | 0.541 |
| CE (22:6) | 5.92 (2.88-12.18) | 6.29 (3.13-12.66) | 0.151 | 0.214 |
| DAG (14:0/18:2) | 0.11 (0.03-0.40) | 0.15 (0.04-0.51) | **<0.001** | **<0.001** |
| DAG (16:0/18:1) | 0.60 (0.18-2.05) | 0.82 (0.24-2.87) | **<0.001** | **<0.001** |
| DAG (16:0/18:2) | 0.88 (0.24-3.23) | 1.27 (0.35-4.63) | **<0.001** | **<0.001** |
| DAG (16:0/18:3) | 0.06 (0.01-0.22) | 0.08 (0.02-0.29) | **<0.001** | **<0.001** |
| DAG (16:0/20:3) | 0.02 (0.01-0.08) | 0.03 (0.01-0.09) | **<0.001** | **<0.001** |
| DAG (16:0/20:4) | 0.07 (0.02-0.29) | 0.09 (0.02-0.39) | **<0.001** | **<0.001** |
| DAG (16:0/22:5) | 0.04 (0.01-0.12) | 0.05 (0.02-0.14) | **<0.001** | **<0.001** |
| DAG (16:0/22:6) | 0.05 (0.01-0.20) | 0.08 (0.02-0.29) | **<0.001** | **<0.001** |
| DAG (16:1/18:2) | 0.25 (0.08-0.81) | 0.33 (0.10-1.06) | **<0.001** | **<0.001** |
| DAG (16:1/18:3) | 0.05 (0.02-0.17) | 0.06 (0.02-0.21) | **<0.001** | **<0.001** |
| DAG (16:1/20:4) | 0.06 (0.01-0.27) | 0.07 (0.02-0.31) | **<0.001** | **<0.001** |
| DAG (18:0/18:1) | 0.57 (0.19-1.75) | 0.76 (0.24-2.40) | **<0.001** | **<0.001** |
| DAG (18:0/18:2) | 0.29 (0.09-0.98) | 0.40 (0.11-1.46) | **<0.001** | **<0.001** |
| DAG (18:1/18:1) | 2.14 (0.68-6.78) | 2.83 (0.89-8.99) | **<0.001** | **<0.001** |
| DAG (18:1/18:2) | 2.39 (0.83-6.87) | 3.12 (1.07-9.09) | **<0.001** | **<0.001** |
| DAG (18:1/20:4) | 0.59 (0.19-1.80) | 0.69 (0.22-2.16) | **<0.001** | **<0.001** |
| DAG (18:1/20:5) | 0.09 (0.02-0.32) | 0.11 (0.03-0.42) | **<0.001** | **<0.001** |
| DAG (18:1/22:5) | 0.07 (0.03-0.22) | 0.09 (0.03-0.28) | **<0.001** | **<0.001** |
| DAG (18:1/22:6) | 0.16 (0.04-0.62) | 0.24 (0.06-0.89) | **<0.001** | **<0.001** |
| DAG (18:2/18:3) | 0.51 (0.13-1.92) | 0.68 (0.18-2.52) | **<0.001** | **<0.001** |
| DAG (18:2/20:3) | 0.04 (0.01-0.11) | 0.05 (0.02-0.13) | **<0.001** | **<0.001** |
| DAG (18:2/20:4) | 0.32 (0.10-1.03) | 0.38 (0.12-1.24) | **<0.001** | **<0.001** |
| DAG (18:2/20:5) | 0.16 (0.05-0.56) | 0.20 (0.06-0.67) | **<0.001** | **<0.001** |
| DAG (18:2/22:5) | 0.04 (0.01-0.12) | 0.05 (0.02-0.16) | **<0.001** | **<0.001** |
| DAG (18:2/22:6) | 0.10 (0.02-0.39) | 0.15 (0.04-0.55) | **<0.001** | **<0.001** |
| TAG (44:3) | 0.03 (0.01-0.12) | 0.04 (0.01-0.15) | **<0.001** | **<0.001** |
| TAG (46:0) | 16.94 (1.18-243.93) | 30.86 (2.60-366.88) | **<0.001** | **<0.001** |
| TAG (46:1) | 19.14 (1.74-210.21) | 31.18 (3.42-284.56) | **<0.001** | **<0.001** |
| TAG (46:2) | 0.89 (0.16-4.83) | 1.24 (0.28-5.52) | **0.006** | **0.013** |
| TAG (46:3) | 0.44 (0.12-1.62) | 0.57 (0.18-1.77) | **0.006** | **0.013** |
| TAG (47:2) | 0.14 (0.04-0.50) | 0.18 (0.06-0.58) | **<0.001** | **<0.001** |
| TAG (48:0) | 107.79 (10.70-1086.24) | 186.43 (21.35-1628.04) | **<0.001** | **<0.001** |
| TAG (48:1) | 161.31 (18.11-1437.22) | 266.50 (36.19-1962.38) | **<0.001** | **<0.001** |
| TAG (48:2) | 8.58 (1.50-49.25) | 12.89 (2.77-59.91) | **<0.001** | **<0.001** |
| TAG (48:3) | 3.94 (0.77-20.08) | 5.55 (1.37-22.52) | **<0.001** | **<0.001** |
| TAG (48:4) | 0.46 (0.11-1.94) | 0.59 (0.18-1.98) | 0.054 | 0.086 |
| TAG (48:5) | 0.13 (0.03-0.54) | 0.16 (0.04-0.59) | 0.099 | 0.148 |
| TAG (49:1) | 21.26 (3.48-130.03) | 31.95 (6.20-164.77) | **<0.001** | **<0.001** |
| TAG (49:2) | 1.27 (0.32-5.07) | 1.78 (0.53-5.99) | **<0.001** | **<0.001** |
| TAG (49:3) | 0.47 (0.13-1.64) | 0.63 (0.21-1.91) | **<0.001** | **<0.001** |
| TAG (50:0) | 25.28 (3.40-188.08) | 42.56 (6.30-287.60) | **<0.001** | **<0.001** |
| TAG (50:1) | 789.51 (169.41-3679.38) | 1130.65 (282.66-4522.67) | **<0.001** | **<0.001** |
| TAG (50:2) | 69.46 (21.41-225.33) | 93.74 (32.93-266.86) | **<0.001** | **<0.001** |
| TAG (50:3) | 49.42 (16.41-148.87) | 65.96 (24.82-175.29) | **<0.001** | **<0.001** |
| TAG (50:4) | 7.38 (2.24-24.38) | 9.78 (3.40-28.12) | **<0.001** | **<0.001** |
| TAG (50:5) | 2.57 (0.62-10.70) | 3.47 (0.95-12.67) | **<0.001** | **<0.001** |
| TAG (50:6) | 0.01 (0.00-0.07) | 0.02 (0.00-0.07) | 0.125 | 0.180 |
| TAG (51:1) | 28.06 (5.05-155.84) | 42.05 (8.66-204.11) | **<0.001** | **<0.001** |
| TAG (51:2) | 4.81 (1.72-13.45) | 6.25 (2.37-16.43) | **<0.001** | **<0.001** |
| TAG (51:3) | 3.27 (1.31-8.13) | 4.19 (1.79-9.83) | **<0.001** | **<0.001** |
| TAG (51:4) | 6.25 (4.87-8.01) | 6.40 (4.99-8.21) | 0.148 | 0.212 |
| TAG (51:5) | 1.22 (0.84-1.78) | 1.32 (0.89-1.95) | **<0.001** | **<0.001** |
| TAG (52:0) | 3.50 (0.58-21.10) | 5.21 (0.72-37.95) | **<0.001** | **<0.001** |
| TAG (52:1) | 51.51 (12.58-210.91) | 75.14 (19.71-286.44) | **<0.001** | **<0.001** |
| TAG (52:2) | 100.32 (43.94-229.03) | 123.62 (54.78-278.94) | **<0.001** | **<0.001** |
| TAG (52:3) | 180.47 (100.76-323.25) | 209.62 (113.15-388.33) | **<0.001** | **<0.001** |
| TAG (52:4) | 73.33 (35.80-150.22) | 88.56 (43.07-182.12) | **<0.001** | **<0.001** |
| TAG (52:5) | 54.98 (17.68-170.96) | 73.01 (25.18-211.67) | **<0.001** | **<0.001** |
| TAG (52:6) | 8.43 (2.29-31.00) | 11.31 (3.36-38.08) | **<0.001** | **<0.001** |
| TAG (52:7) | 0.37 (0.08-1.76) | 0.55 (0.13-2.34) | **<0.001** | **<0.001** |
| TAG (52:8) | 0.20 (0.04-0.97) | 0.30 (0.07-1.31) | **<0.001** | **<0.001** |
| TAG (53:0) | 0.12 (0.03-0.45) | 0.16 (0.04-0.62) | **<0.001** | **<0.001** |
| TAG (53:1) | 1.79 (0.44-7.24) | 2.54 (0.69-9.43) | **<0.001** | **<0.001** |
| TAG (53:2) | 5.13 (1.92-13.71) | 6.54 (2.60-16.40) | **<0.001** | **<0.001** |
| TAG (53:3) | 4.51 (1.66-12.23) | 5.83 (2.28-14.90) | **<0.001** | **<0.001** |
| TAG (53:4) | 2.68 (1.17-6.13) | 3.26 (1.43-7.43) | **<0.001** | **<0.001** |
| TAG (53:5) | 0.05 (0.01-0.21) | 0.07 (0.02-0.24) | **<0.001** | **<0.001** |
| TAG (53:6) | 0.036 (0.011-0.118) | 0.044 (0.015-0.133) | **<0.001** | **<0.001** |
| TAG (54:1) | 9.68 (2.22-42.27) | 14.24 (3.26-62.18) | **<0.001** | **<0.001** |
| TAG (54:2) | 30.44 (9.90-93.60) | 40.73 (13.75-120.66) | **<0.001** | **<0.001** |
| TAG (54:3) | 83.14 (41.27-167.46) | 99.90 (50.69-196.87) | **<0.001** | **<0.001** |
| TAG (54:4) | 153.29 (67.12-350.10) | 186.43 (81.27-427.65) | **<0.001** | **<0.001** |
| TAG (54:5) | 94.29 (39.74-223.70) | 113.94 (48.53-267.54) | **<0.001** | **<0.001** |
| TAG (54:6) | 58.23 (21.08-160.83) | 71.98 (27.03-191.62) | **<0.001** | **<0.001** |
| TAG (54:7) | 20.09 (5.91-68.29) | 25.60 (8.22-79.67) | **<0.001** | **<0.001** |
| TAG (54:8) | 2.65 (0.67-10.39) | 3.39 (0.90-12.75) | **<0.001** | **<0.001** |
| TAG (55:1) | 0.88 (0.33-2.33) | 1.12 (0.44-2.86) | **<0.001** | **<0.001** |
| TAG (55:2) | 0.50 (0.19-1.33) | 0.58 (0.22-1.54) | **<0.001** | **0.002** |
| TAG (55:3) | 0.42 (0.16-1.06) | 0.50 (0.20-1.26) | **<0.001** | **0.001** |
| TAG (55:4) | 0.28 (0.10-0.77) | 0.33 (0.12-0.90) | **0.017** | **0.031** |
| TAG (55:5) | 0.68 (0.25-1.83) | 0.80 (0.30-2.12) | **0.008** | **0.016** |
| TAG (55:7) | 0.03 (0.01-0.12) | 0.05 (0.01-0.17) | **<0.001** | **<0.001** |
| TAG (56:1) | 0.53 (0.11-2.58) | 0.72 (0.14-3.68) | **0.003** | **0.007** |
| TAG (56:10) | 0.06 (0.02-0.19) | 0.08 (0.02-0.24) | **<0.001** | **<0.001** |
| TAG (56:2) | 1.06 (0.27-4.27) | 1.32 (0.34-5.12) | 0.053 | 0.085 |
| TAG (56:3) | 4.79 (1.49-15.38) | 5.63 (1.88-16.82) | 0.12 | 0.17 |
| TAG (56:4) | 11.37 (4.00-32.35) | 13.96 (5.04-38.67) | **<0.001** | **<0.001** |
| TAG (56:5) | 15.9 (6.46-39.29) | 19.25 (7.97-46.52) | **<0.001** | **<0.001** |
| TAG (56:6) | 24.5 (10.3-58.3) | 29.70 (12.76-69.11) | **<0.001** | **<0.001** |
| TAG (56:7) | 26.0 (10.6-63.4) | 32.5 (13.4-78.8) | **<0.001** | **<0.001** |
| TAG (56:8) | 14.9 (5.49-40.5) | 19.3 (7.16-52.1) | **<0.001** | **<0.001** |
| TAG (56:9) | 3.38 (1.15-9.89) | 4.23 (1.4-12.5) | **<0.001** | **<0.001** |
| TAG (57:2) | 0.10 (0.04-0.28) | 0.13 (0.04-0.36) | **<0.001** | **<0.001** |
| TAG (57:3) | 0.30 (0.11-0.81) | 0.37 (0.13-1.01) | **0.001** | **0.003** |
| TAG (58:10) | 2.90 (0.93-9.06) | 3.68 (1.17-11.54) | **<0.001** | **<0.001** |
| TAG (58:3) | 0.05 (0.01-0.52) | 0.07 (0.01-0.56) | 0.754 | 0.802 |
| TAG (58:5) | 0.04 (0.01-0.15) | 0.05 (0.01-0.19) | **0.008** | **0.015** |
| TAG (58:6) | 1.66 (0.63-4.36) | 2.04 (0.78-5.32) | **<0.001** | **<0.001** |
| TAG (58:7) | 4.18 (1.68-10.39) | 5.21 (2.06-13.17) | **<0.001** | **<0.001** |
| TAG (58:8) | 5.01 (1.92-13.07) | 6.36 (2.37-17.10) | **<0.001** | **<0.001** |
| TAG (58:9) | 4.84 (1.73-13.54) | 6.16 (2.15-17.70) | **<0.001** | **<0.001** |
| TAG (60:10) | 0.54 (0.18-1.59) | 0.64 (0.22-1.82) | **<0.001** | **<0.001** |
| TAG (60:11) | 0.36 (0.10-1.37) | 0.49 (0.13-1.87) | **<0.001** | **<0.001** |
| TAG (60:12) | 0.04 (0.01-0.16) | 0.05 (0.01-0.22) | **<0.001** | **<0.001** |

Values were the geometric mean (95% CI). The *p*-values were calculated after adjustment for age, sex, region, and residence, and corrected for multiple hypothesis testing using the Benjamini-Hochberg method. Statistically significant *p*-values and FDR (<0.05) are indicated in boldface type. Abbreviations: CE, cholesteryl ester; Cer, ceramide; DAG, diacylglycerol; dhCer, dihydroceramide; FDR, false-discovery rate; GluCer, glucosylceramide; HexCer, hexosylceramide; LacCer, lactosylceramide; LPC, lysophosphatidylcholine; LPI, lysophosphatidylinositol; PC, phosphatidylcholine; PE, phosphatidylethanolamine, PE-O, alkylphosphatidylethanolamine, PE-P, alkenylphosphatidylethanolamine; PS, phosphatidylserine; SM, sphingomyelin; SM (OH), hydroxyl-sphingomyelin (with one additional hydroxyl); SM (2OH), hydroxyl-sphingomyelin (with two additional hydroxyls); TAG, triacylglycerol.

# Table S3 Sensitivity analyses by excluding participants with lipid-lowering medication use and declined kidney function

| **Significant lipids with hyperuricemia** | **RR(95% CI)** | ***p*** | **FDR** |
| --- | --- | --- | --- |
| DAG (16:0/22:5) | 1.35 (1.10 - 1.66) | 0.004 | 0.04 |
| DAG (18:1/20:5) | 1.41 (1.18 - 1.69) | <0.001 | 0.008 |
| DAG (16:0/22:6) | 1.51 (1.24 - 1.83) | 0.009 | 0.003 |
| DAG (18:1/22:6) | 1.49 (1.25 - 1.79) | <0.001 | 0.003 |
| TAG (53:0) | 1.53 (1.25 - 1.86) | <0.001 | 0.003 |
| PC (16:0/20:5) | 1.40 (1.19 - 1.64) | <0.001 | 0.002 |
| LPC (20:2) | 0.79 (0.66 - 0.95) | 0.010 | 0.07 |

*p-*values were corrected for multiple hypothesis testing using the Benjamini-Hochberg method. Statistically significant *p-*values and FDR (<0.05) are indicated in boldface type. Model was adjusted for demographic (sex, age, region, residence, education), and lifestyle (smoking status, drinking status, physical activity), metabolic traits (body mass index, γ-glutamyl transpeptidase, creatinine, homeostatic model assessment of insulin resistance, total triglycerides, and total cholesterol, hypertension, family history of chronic diseases, and medication status (antihypertensive medicines).

**Table S4 Relative risks of hyperuricemia per SD increment of significant lipids in stratified analysis**

|  | **n** | **PC (16:0/20:5)** | ***p*** | **LPC (20:2)** | ***p*** | **DAG (16:0/22:5)** | ***p*** | **DAG (18:1/20:5)** | ***p*** |
| --- | --- | --- | --- | --- | --- | --- | --- | --- | --- |
| **Age, year** |  |  |  |  |  |  |  |  |  |
| 50-59 | 1321 | 1.31 (1.05-1.64) | 0.02 | 0.7 (0.55-0.9) | 0.004 | 1.5 (1.15-1.96) | 0.003 | 1.37 (1.08-1.74) | 0.009 |
| 60-70 | 926 | 1.26 (1.03-1.55) | 0.03 | 0.88 (0.72-1.08) | 0.23 | 1.2 (0.96-1.51) | 0.12 | 1.2 (0.96-1.49) | 0.11 |
|  |  |  | *0.91* |  | *0.70* |  | *0.76* |  | *0.79* |
| **Sex** |  |  |  |  |  |  |  |  |  |
| Men | 950 | 1.32 (1.07-1.63) | 0.009 | 0.88 (0.72-1.07) | 0.19 | 1.27 (1-1.6) | 0.04 | 1.35 (1.08-1.69) | 0.008 |
| Women | 1297 | 1.27 (1.02-1.58) | 0.04 | 0.63 (0.49-0.82) | 0.001 | 1.47 (1.13-1.91) | 0.004 | 1.2 (0.95-1.52) | 0.13 |
|  |  |  | *0.91* |  | *0.63* |  | *0.91* |  | *0.91* |
| **Region** |  |  |  |  |  |  |  |  |  |
| North | 1114 | 1.16 (0.91-1.49) | 0.24 | 0.86 (0.67-1.12) | 0.27 | 1.6 (1.19-2.14) | 0.002 | 1.25 (0.95-1.65) | 0.12 |
| South | 1133 | 1.36 (1.12-1.64) | 0.002 | 0.75 (0.61-0.92) | 0.006 | 1.27 (1.03-1.58) | 0.03 | 1.32 (1.08-1.6) | 0.006 |
|  |  |  | *0.76* |  | *0.80* |  | *0.79* |  | *0.88* |
| **Residence** |  |  |  |  |  |  |  |  |  |
| Rural | 1283 | 1.38 (1.06-1.78) | 0.02 | 0.72 (0.56-0.93) | 0.01 | 1.45 (1.08-1.95) | 0.01 | 1.43 (1.08-1.88) | 0.01 |
| Urban | 964 | 1.25 (1.03-1.51) | 0.02 | 0.83 (0.67-1.02) | 0.08 | 1.3 (1.04-1.62) | 0.02 | 1.21 (0.99-1.48) | 0.07 |
|  |  |  | *0.63* |  | *0.76* |  | *0.78* |  | *0.63* |
| **Current smoking** |  |  |  |  |  |  |  |  |  |
| Yes | 614 | 1.27 (1.07-1.52) | 0.007 | 0.72 (0.59-0.88) | 0.001 | 1.38 (1.12-1.69) | 0.002 | 1.25 (1.04-1.5) | 0.02 |
| No | 1633 | 1.29 (0.97-1.73) | 0.08 | 0.87 (0.67-1.14) | 0.31 | 1.26 (0.89-1.76) | 0.19 | 1.32 (0.97-1.8) | 0.08 |
|  |  |  | *0.91* |  | *0.65* |  | *0.91* |  | *0.86* |
| **Current drinking** |  |  |  |  |  |  |  |  |  |
| Yes | 562 | 1.33 (1.12-1.57) | 0.001 | 0.68 (0.56-0.82) | <0.001 | 1.44 (1.19-1.75) | <0.001 | 1.34 (1.13-1.6) | 0.001 |
| No | 1685 | 1.45 (0.99-2.11) | 0.05 | 1 (0.73-1.35) | 0.99 | 1.48 (1.01-2.18) | 0.05 | 1.44 (0.97-2.12) | 0.07 |
|  |  |  | *0.82* |  | *0.63* |  | *0.78* |  | *0.77* |
| **Hypertension** |  |  |  |  |  |  |  |  |  |
| Yes | 1545 | 1.26 (1.07-1.5) | 0.007 | 0.79 (0.66-0.95) | 0.01 | 1.39 (1.15-1.68) | 0.001 | 1.25 (1.04-1.49) | 0.02 |
| No | 702 | 1.36 (0.97-1.91) | 0.07 | 0.66 (0.45-0.95) | 0.025 | 1.33 (0.87-2.03) | 0.182 | 1.48 (1.02-2.15) | 0.04 |
|  |  |  | *0.91* |  | *0.91* |  | *0.91* |  | *0.91* |
| **BMI, kg/m^2^** |  |  |  |  |  |  |  |  |  |
| ≥24 | 1209 | 1.25 (1.05-1.49) | 0.01 | 0.81 (0.67-0.98) | 0.03 | 1.34 (1.10-1.64) | 0.004 | 1.27 (1.06-1.53) | 0.01 |
| <24 | 1038 | 1.44 (1.08-1.91) | 0.01 | 0.65 (0.48-0.86) | 0.003 | 1.79 (1.26-2.53) | 0.001 | 1.48 (1.08-2.03) | 0.02 |
|  |  |  | *0.50* |  | *0.34* |  | *0.86* |  | *0.65* |
| **HOMA-IR** |  |  |  |  |  |  |  |  |  |
| ≥1.79 | 1123 | 1.16 (0.99-1.37) | 0.074 | 0.87 (0.74-1.02) | 0.095 | 1.42 (1.19-1.69) | <0.001 | 1.27 (1.08-1.50) | 0.003 |
| <1.79 | 1121 | 1.43 (1.16-1.76) | 0.001 | 0.71 (0.59-0.85) | <0.001 | 1.45 (0.58-1.62) | 0.42 | 1.89 (1.16-2.22) | 0.02 |
|  |  |  | *0.74* |  | *0.79* |  | *0.63* |  | *0.65* |
| **Triglycerides, mmol/L** |  |  |  |  |  |  |  |  |  |
| ≥1.7 | 530 | 1.13 (0.88-1.46) | 0.35 | 0.74 (0.57-0.96) | 0.02 | 1.26 (0.97-1.63) | 0.08 | 1.12 (0.89-1.41) | 0.33 |
| <1.7 | 1717 | 1.36 (1.13-1.63) | 0.001 | 0.85 (0.7-1.03) | 0.10 | 1.49 (1.21-1.84) | <0.001 | 1.47 (1.2-1.81) | <0.001 |
|  |  |  | *0.91* |  | *0.91* |  | *0.93* |  | *0.91* |
| **Total cholesterol, mmol/L** |  |  |  |  |  |  |  |  |  |
| ≥4.68 | 1054 | 1.28 (1.05-1.56) | 0.02 | 0.80 (0.65-0.99) | 0.04 | 1.37 (1.08-1.74) | 0.008 | 1.26 (1.02-1.55) | 0.03 |
| <4.68 | 1193 | 1.21 (0.96-1.53) | 0.10 | 0.78 (0.61-0.99) | 0.04 | 1.31 (1.00-1.72) | 0.05 | 1.28 (0.99-1.67) | 0.06 |
|  |  |  | *0.91* |  | *0.91* |  | *0.93* |  | *0.91* |
| **Creatinine, mg/dL** |  |  |  |  |  |  |  |  |  |
| ≥54 | 1169 | 1.23 (1.05-1.44) | 0.01 | 0.87 (0.74-1.03) | 0.12 | 1.28 (1.06-1.53) | 0.008 | 1.26 (1.06-1.49) | 0.009 |
| <54 | 1078 | 1.17 (0.80-1.70) | 0.42 | 0.50 (0.31-0.8) | 0.004 | 1.28 (0.81-2.02) | 0.29 | 0.93 (0.62-1.37) | 0.70 |
|  |  |  | *0.78* |  | *0.63* |  | *0.65* |  | *0.8* |
| **GGT, U/L** |  |  |  |  |  |  |  |  |  |
| ≥24 | 1140 | 1.20 (1.01-1.42) | 0.04 | 0.86 (0.72-1.03) | 0.10 | 1.25 (1.02-1.53) | 0.03 | 1.17 (0.97-1.41) | 0.09 |
| <24 | 1107 | 1.55 (1.13-2.13) | 0.006 | 0.45 (0.31-0.66) | <0.001 | 1.34 (0.94-1.92) | 0.11 | 1.42 (1.01-2.02) | 0.05 |
|  |  |  | *0.65* |  | *0.78* |  | *0.91* |  | *0.91* |
| **Antihypertensive medicine** |  |  |  |  |  |  |  |  |  |
| Yes | 590 | 1.20 (0.97-1.49) | 0.10 | 0.75 (0.58-0.96) | 0.02 | 1.39 (1.09-1.78) | 0.009 | 1.2 (0.96-1.51) | 0.114 |
| No | 1657 | 1.34 (1.08-1.64) | 0.006 | 0.80 (0.65-0.99) | 0.03 | 1.37 (1.08-1.73) | 0.009 | 1.35 (1.07-1.69) | 0.01 |
|  |  |  | *0.91* |  | *0.86* |  | *0.86* |  | *0.86* |
| **Lipid-lowering medicine** |  |  |  |  |  |  |  |  |  |
| Yes | 2106 | 1.36 (1.16-1.59) | <0.001 | 0.79 (0.66-0.93) | 0.005 | 1.46 (1.22-1.76) | <0.001 | 1.41 (1.19-1.68) | <0.001 |
| No | 141 | 1.05 (0.62-1.78) | 0.86 | 0.46 (0.25-0.84) | 0.01 | 1.24 (0.68-2.28) | 0.48 | 0.84 (0.46-1.51) | 0.55 |
|  |  |  | *0.65* |  | *0.74* |  | *0.79* |  | *0.65* |
| **Declined kidney function** |  |  |  |  |  |  |  |  |  |
| Yes | 50 | 1.13 (0.51-2.48) | 0.77 | 0.97 (0.65-1.45) | 0.88 | 0.94 (0.42-2.13) | 0.89 | 1.08 (0.49-2.38) | 0.85 |
| No | 2197 | 1.36 (1.17-1.59) | <0.001 | 0.78 (0.65-0.92) | 0.003 | 1.45 (1.22-1.73) | <0.001 | 1.32 (1.12-1.56) | 0.001 |
|  |  |  | *0.65* |  | *0.8* |  | *0.13* |  | *0.86* |
| **Cardiovascular diseases** |  |  |  |  |  |  |  |  |  |
| No | 1993 | 1.41 (1.2-1.66) | <0.001 | 0.75 (0.63-0.9) | 0.002 | 1.36 (1.13-1.64) | 0.001 | 1.37 (1.15-1.62) | <0.001 |
| Yes | 254 | 0.97 (0.68-1.39) | 0.88 | 0.90 (0.62-1.31) | 0.57 | 1.45 (0.95-2.22) | 0.08 | 1.14 (0.75-1.71) | 0.54 |
|  |  |  | *0.65* |  | *0.91* |  | *0.91* |  | *0.84* |
| **Type 2 diabetes** |  |  |  |  |  |  |  |  |  |
| No | 1973 | 1.33 (1.14-1.55) | <0.001 | 0.77 (0.65-0.91) | 0.002 | 1.42 (1.19-1.69) | <0.001 | 1.35 (1.14-1.6) | <0.001 |
| Yes | 274 | 1.38 (0.79-2.43) | 0.25 | 0.84 (0.48-1.47) | 0.54 | 2.45 (1.3-4.59) | 0.005 | 1.64 (0.93-2.89) | 0.08 |
|  |  |  | *0.78* |  | *0.91* |  | *0.65* |  | *0.65* |

(continuted)

|  | **CE (20:2)** | ***p*** | **DAG (16:0/22:6)** | ***p*** | **TAG (53:0)** | ***p*** |
| --- | --- | --- | --- | --- | --- | --- |
| **Age, year** |  |  |  |  |  |  |
| 50-59 | 1.44 (1.11-1.86) | 0.006 | 1.39 (1.1-1.76) | 0.005 | 1.44 (1.10-1.89) | 0.007 |
| 60-70 | 1.28 (1.01-1.61) | 0.04 | 1.22 (0.97-1.53) | 0.08 | 1.24 (0.98-1.55) | 0.07 |
|  |  | *0.79* |  | *0.8* |  | *0.80* |
| **Sex** |  |  |  |  |  |  |
| Men | 1.38 (1.09-1.74) | 0.007 | 1.38 (1.11-1.73) | 0.004 | 1.44 (1.13-1.83) | 0.003 |
| Women | 1.41 (1.07-1.85) | 0.01 | 1.22 (0.94-1.58) | 0.13 | 1.24 (0.95-1.62) | 0.12 |
|  |  | *0.91* |  | *0.84* |  | *0.86* |
| **Region** |  |  |  |  |  |  |
| North | 1.49 (1.11-2) | 0.008 | 1.4 (1.05-1.87) | 0.022 | 1.49 (1.11-2.01) | 0.008 |
| South | 1.33 (1.08-1.64) | 0.006 | 1.31 (1.07-1.59) | 0.008 | 1.37 (1.1-1.7) | 0.004 |
|  |  | *0.78* |  | *0.80* |  | *0.91* |
| **Residence** |  |  |  |  |  |  |
| Rural | 1.63 (1.21-2.19) | 0.001 | 1.62 (1.22-2.16) | 0.001 | 1.38 (1.01-1.86) | 0.04 |
| Urban | 1.3 (1.05-1.61) | 0.02 | 1.20 (0.98-1.47) | 0.07 | 1.36 (1.09-1.7) | 0.006 |
|  |  | *0.65* |  | *0.63* |  | *0.76* |
| **Current smoking** |  |  |  |  |  |  |
| Yes | 1.36 (1.12-1.67) | 0.003 | 1.29 (1.06-1.57) | 0.01 | 1.27 (1.04-1.55) | 0.02 |
| No | 1.33 (0.95-1.85) | 0.10 | 1.30 (0.96-1.76) | 0.09 | 1.52 (1.06-2.17) | 0.02 |
|  |  | *0.91* |  | *0.85* |  | *0.78* |
| **Current drinking** |  |  |  |  |  |  |
| Yes | 1.45 (1.2-1.75) | <0.001 | 1.36 (1.13-1.63) | 0.001 | 1.41 (1.16-1.70) | <0.001 |
| No | 1.67 (1.12-2.49) | 0.01 | 1.78 (1.23-2.57) | 0.002 | 1.49 (0.99-2.25) | 0.05 |
|  |  | *0.78* |  | *0.91* |  | *0.78* |
| **Hypertension** |  |  |  |  |  |  |
| Yes | 1.41 (1.17-1.71) | <0.001 | 1.3 (1.08-1.56) | 0.006 | 1.33 (1.10-1.62) | 0.004 |
| No | 1.3 (0.88-1.91) | 0.19 | 1.59 (1.11-2.26) | 0.01 | 1.37 (0.91-2.07) | 0.13 |
|  |  | *0.91* |  | *0.84* |  | *0.91* |
| **BMI, kg/m^2^** |  |  |  |  |  |  |
| ≥24 | 1.26 (1.04-1.53) | 0.02 | 1.19 (0.98-1.44) | 0.08 | 1.35 (1.11-1.65) | 0.003 |
| <24 | 2.16 (1.55-3.02) | <0.001 | 2.19 (1.62-2.96) | <0.001 | 1.72 (1.21-2.44) | 0.002 |
|  |  | *0.65* |  | *0.50* |  | *0.65* |
| **HOMA-IR** |  |  |  |  |  |  |
| ≥1.79 | 1.27 (1.06-1.51) | 0.008 | 1.23 (1.04-1.46) | 0.017 | 1.29 (1.08-1.53) | 0.005 |
| <1.79 | 1.09 (0.87-1.36) | 0.449 | 1.84 (1.43-2.35) | <0.001 | 1.59 (1.24-2.05) | <0.001 |
|  |  | *0.76* |  | *0.63* |  | *0.65* |
| **Triglycerides, mmol/L** |  |  |  |  |  |  |
| ≥1.7 | 1.32 (1.01-1.74) | 0.04 | 1.25 (0.96-1.64) | 0.10 | 1.2 (0.89-1.60) | 0.23 |
| <1.7 | 1.55 (1.26-1.91) | <0.001 | 1.58 (1.30-1.93) | <0.001 | 1.54 (1.25-1.90) | <0.001 |
|  |  | *0.91* |  | *0.91* |  | *0.91* |
| **Total cholesterol, mmol/L** |  |  |  |  |  |  |
| ≥4.68 | 1.33 (1.06-1.67) | 0.01 | 1.26 (1.02-1.57) | 0.05 | 1.41 (1.11-1.78) | 0.005 |
| <4.68 | 1.43 (1.08-1.88) | 0.01 | 1.44 (1.11-1.88) | 0.007 | 1.30 (1.00-1.7) | 0.05 |
|  |  | *0.91* |  | *0.91* |  | *0.91* |
| **Creatinine, mg/dL** |  |  |  |  |  |  |
| ≥54 | 1.32 (1.11-1.58) | 0.002 | 1.32 (1.12-1.57) | 0.001 | 1.31 (1.09-1.57) | 0.003 |
| <24 | 1.32 (0.83-2.09) | 0.25 | 1.14 (0.73-1.8) | 0.56 | 1.04 (0.66-1.64) | 0.87 |
|  |  | *0.65* |  | *0.63* |  | *0.63* |
| **GGT, U/L** |  |  |  |  |  |  |
| ≥24 | 1.27 (1.04-1.56) | 0.02 | 1.23 (1.02-1.49) | 0.03 | 1.27 (1.04-1.56) | 0.02 |
| <24 | 1.54 (1.10-2.16) | 0.01 | 1.50 (1.08-2.08) | 0.02 | 1.42 (0.99-2.03) | 0.06 |
|  |  | *0.86* |  | *0.86* |  | *0.93* |
| **Antihypertensive medicine** |  |  |  |  |  |  |
| Yes | 1.20 (0.95-1.52) | 0.12 | 1.39 (1.09-1.78) | 0.009 | 1.25 (0.97-1.61) | 0.08 |
| No | 1.12 (0.91-1.39) | 0.28 | 1.37 (1.08-1.73) | 0.009 | 1.42 (1.11-1.80) | 0.004 |
|  |  | *0.91* |  | *0.86* |  | *0.86* |
| **Lipid-lowering medicine** |  |  |  |  |  |  |
| Yes | 1.18 (1.00-1.39) | 0.04 | 1.46 (1.22-1.76) | <0.001 | 1.46 (1.20-1.77) | <0.001 |
| No | 1.64 (0.90-2.99) | 0.11 | 1.24 (0.68-2.28) | 0.48 | 1.01 (0.57-1.79) | 0.96 |
|  |  | *0.79* |  | *0.79* |  | *0.76* |
| **Declined kidney function** |  |  |  |  |  |  |
| Yes | 1.06 (0.55-2.05) | 0.86 | 0.94 (0.42-2.13) | 0.89 | 0.94 (0.49-1.81) | 0.86 |
| No | 1.23 (1.04-1.44) | 0.02 | 1.45 (1.22-1.73) | <0.001 | 1.44 (1.20-1.72) | <0.001 |
|  |  | *0.86* |  | *0.13* |  | *0.63* |
| **Cardiovascular diseases** |  |  |  |  |  |  |
| No | 1.15 (0.97-1.36) | 0.12 | 1.36 (1.13-1.64) | 0.001 | 1.35 (1.12-1.63) | 0.002 |
| Yes | 1.38 (0.94-2.03) | 0.09 | 1.45 (0.95-2.22) | 0.09 | 1.39 (0.89-2.19) | 0.15 |
|  |  | *0.78* |  | *0.91* |  | *0.91* |
| **Type 2 diabetes** |  |  |  |  |  |  |
| No | 1.15 (0.97-1.35) | 0.10 | 1.42 (1.19-1.69) | <0.001 | 1.35 (1.13-1.61) | 0.001 |
| Yes | 1.52 (0.86-2.69) | 0.15 | 2.45 (1.30-4.59) | 0.005 | 2.26 (1.15-4.44) | 0.02 |
|  |  | *0.75* |  | *0.65* |  | *0.78* |

Model was adjusted for demographic (sex, age, region, residence, education) and lifestyle (smoking, drinking, physical activity) information, metabolic traits (BMI, GGT, creatinine, HOMA-IR, triglycerides, and total cholesterol), diseases (hypertension, family history of chronic diseases) and medication status (antihypertensive or lipid-lowering medicines), except stratifying factors. P for interaction has been adjusted for FDR correction using the Benjamini-Hochberg method and is shown in Italics. Abbreviations: BMI, body mass index; CE, cholesteryl ester; DAG, diacylglycerol; GGT, γ-glutamyl transpeptidase; HOMA-IR homeostatic model assessment of insulin resistance; LPC, lysophosphatidylcholine; PC, phosphatidylcholine

# Table S5 Mediation analyses to assess mediation effects of retinol binding protein 4 for the association between significant lipids and hyperuricemia

|  | **Model 1** | | **Model 2** | | |
| --- | --- | --- | --- | --- | --- |
|  | **Mediation proportions (95% CI)** | **FDR** | **Mediation proportion (95% CI)** | **FDR** |  |
| DAG (16:0/22:5) | 0.23 (0.14~0.36) | <0.001 | 0.09 (0.03~0.28) | <0.001 |  |
| DAG (18:1/20:5) | 0.29 (0.17~0.47) | <0.001 | 0.11 (0.04~0.33) | <0.001 |  |
| DAG (16:0/22:6) | 0.22 (0.14~0.30) | <0.001 | 0.12 (0.05~0.39) | <0.001 |  |
| DAG (18:1/22:6) | 0.22 (0.13~0.33) | <0.001 | 0.14 (0.05~0.32) | <0.001 |  |
| TAG (53:0) | 0.25 (0.13~0.42) | <0.001 | 0.14 (0.06~0.36) | <0.001 |  |
| PC (16:0/20:5) | 0.35 (0.18~0.74) | <0.001 | 0.14 (0.04~0.37) | 0.02 |  |
| LPC (20:2) | -0.11 (-0.25~-0.02) | <0.001 | -0.05 (-0.60~0.00) | 0.02 |  |

The mediation effect was estimated by a linear model for retinol binding protein 4 and a log-Poisson regression model for the binary outcome of hyperuricemia. *p-*values were identified by multivariable log-Poisson regression models. FDR values were corrected for multiple testing using the Benjamini-Hochberg method. Model 1: adjusted for age, sex, region, residence, educational attainment, physical activity, smoking status, drinking status, and family history of chronic diseases; Model 2: further adjusted for body mass index, γ-glutamyl transpeptidase, creatinine, homeostatic model assessment of insulin resistance, hypertension, medication status (antihypertensive or lipid-lowering medicines), total triglycerides and total cholesterol. Abbreviations: DAG, diacylglycerol; LPC, lysophosphatidylcholine; PC, phosphatidylcholine; TAG, triacylglycerol

# Table S6 Network module information and association between modules and uric acid/hyperuricemia

| **Module** | **Composition** | **Description** | **Uric acid (μmol/L)** | | | | **Hyperuricemia** | | | |
| --- | --- | --- | --- | --- | --- | --- | --- | --- | --- | --- |
|  |  |  | **Model1 (β, 95% CI)** | **FDR** | **Model2 (β, 95% CI)** | **FDR** | **Model1 (RR, 95% CI)** | **FDR** | **Model2 (RR, 95% CI)** | **FDR** |
| **MEbrown** | **34 (18 TAGs, 8 PCs, 6 DAGs, 2 CEs)** | **For TAGs, carbon n=53~60, double bonds=0~12; for PCs, carbon n=36~40, double bonds=5~8; for DAGs, carbon n=38~40, double bonds=6~8; CE (22:0) and CE (22:6).**  **For PC, PE, PS, DAG subclasses, most lipids containing SFA or MUFA acyl chain in sn-1 position.** | **14.0 (10.9,17.1)** | **<0.001** | **7.24 (4.03,10.44)** | **<0.001** | **1.41 (1.27,1.56)** | **<0.001** | **1.27 (1.11,1.45)** | **0.04** |
| **MEyellow** | **32 (23 TAGs, 7 DAGs, 2 LPCs)** | **For TAGs, carbon n=51~58, double bonds=2~10; for DAGs, carbon n=34~38, double bonds=2~7; LPC (16:0) and LPC (18:0)** | **14.42 (11.14,17.7)** | **<0.001** | **2.28 (-2.43,7)** | **0.59** | **1.4 (1.27,1.55)** | **<0.001** | **1.13 (0.95,1.33)** | **0.47** |
| **MEblue** | **39 (37 PEs, 2 PSs)** | **For PEs, carbon n=32~40, double bonds=0~8; PS (18:0/18:0) and PS (18:0/18:1)** | **9.84 (6.65,13.0)** | **<0.001** | **0.59 (-2.73,3.90)** | **0.74** | **1.37 (1.22,1.53)** | **<0.001** | **1.1 (0.96,1.26)** | **0.47** |
| **MEturquoise** | **60 (38 TAGs, 12 DAGs, 8 PCs, 1 PE, 1 CE)** | **For TAGs, carbon n=44~58, double bonds=0~6; for DAGs, carbon n=32~38, double bonds=1~5; for PCs, carbon n=32~36, double bonds=0~5; PE (16:0/20:1); CE (18:0)** | **15.92 (12.9,19.0)** | **<0.001** | **9.02 (4.95,13.09)** | **<0.001** | **1.48 (1.34,1.65)** | **<0.001** | **1.28 (1.09,1.51)** | **0.11** |
| MEsalmon | 11 PEs | For PEs, carbon n=36~40, double bonds=0,5~8 | 4.91 (1.44,8.37) | 0.007 | 4.06 (1.22,6.90) | 0.03 | 1.15 (1.01,1.31) | 0.084 | 1.15 (1.02,1.31) | 0.29 |
| MEcyan | 10 SMs | For SMs, carbon n=38~42, 74, 78, 82. | 6.34 (3.14,9.53) | <0.001 | 2.60 (-0.14,5.35) | 0.22 | 1.12 (1.00,1.26) | 0.14 | 1.07 (0.94,1.23) | 0.59 |
| MEgreenyellow | 11 (8 HexCers, 2 GlcCers, 1 Cer) | For HexCers, carbon n=16~24, double bonds=0, 1; GlcCer (d18:0/24:0), GlcCer (d18:0/24:1) and Cer (26:0) | -0.84 (-4.12,2.45) | 0.66 | -1.22 (-3.94,1.49) | 0.62 | 0.99 (0.86,1.14) | 0.89 | 1.02 (0.91,1.14) | 0.83 |
| MEblack | 18 (10 Pes, 3 Cers, 3 dhCers, 1 LacCer, 1 SM) | For PEs, carbon n=38, 40, double bonds=4,5,6; for Cers, carbon n=22, 24, double bonds=0,1; for dhCers, carbon n=22, 24, double bonds=0, 1; LacCer (20:1) and SM (40:4;3) | 7.57 (3.26,11.9) | <0.001 | 1.07 (-2.57,4.71) | 0.67 | 1.17 (1.01,1.35) | 0.092 | 1.03 (0.9,1.19) | 0.83 |
| MEgrey | 14 (12 PEs, 1 PS, 1 PC) | For Pes, carbon n = 36~40, double bonds=3~6; for PS, carbon n= 40, double bonds=4; for PS, carbon n=38, double bonds=6 | 2.06 (-1.52,5.63) | 0.31 | 1.21 (-1.77,4.18) | 0.62 | 1.07 (0.94,1.22) | 0.45 | 1.06 (0.93,1.2) | 0.62 |
| **MEviolet** | **17 (7 SMs, 3 dhCers, 2 Cers, 2 LPCs, 2 PCs, 1 LPC)** | **For SMs, carbon n=38~44, double bonds=0~3; for dhCers, carbon n=18, 20, double bonds=0,1; for Cers, carbon n=14, 26, double bonds=0,1; for LPCs, carbon n=18, 20, double bonds=1,2; for PCs, carbon n=38, double bonds=2,3; LPI (18:1)** | **-8.87 (-12.1,-5.69)** | **<0.001** | **-3.76 (-6.65,-0.86)** | **0.03** | **0.78 (0.68,0.91)** | **0.004** | **0.89 (0.76,1.04)** | **0.44** |
| MEtan | 11 (5 PCs, 4 CEs, 1 LPC, 1 SM) | For PCs, carbon n=38, 40, 44, double bonds=4~7; for CEs, carbon n=20, double bonds=2~5; | 0.26 (-3.03,3.56) | 0.88 | -0.52 (-3.25,2.2) | 0.74 | 1.03 (0.91,1.17) | 0.692 | 1.05 (0.93,1.19) | 0.62 |
| MEgreen | 31 (19 SMs, 6 Cers, 3 dhCers, 1 HexCer, 1 LPC, 1 PC) | For SMs, carbon n=30~80, double bonds=0~6; for Cers, carbon n=16~24, double bonds=0,1; for dhCers, carbon n=16~24, double bonds=0,1; LPC (20:0); HexCer (12:0); PC (18:2/16:1) | 3.80 (0.26,7.34) | 0.04 | -0.86 (-3.84,2.11) | 0.69 | 1.15 (1.01,1.31) | 0.084 | 1.06 (0.94,1.20) | 0.59 |
| **MEpink** | **18 (14 PCs, 2 LPCs, 2 SMs)** | **For PCs, carbon n=20~42, double bonds=2~6; for LPCs, carbon n=20, double bonds=2,3; for SMs, carbon n=42, 44, double bonds=3, 4** | **8.35 (5.04,11.7)** | **<0.001** | **2.42 (-0.69,5.53)** | **0.31** | **1.28 (1.13,1.45)** | **0.001** | **1.13 (0.99,1.29)** | **0.44** |
| MEred | 29 (20 PEs, 5 PCs, 3 CEs, 1 SM) | For PEs, carbon n=34~38, double bonds=1~4; for PCs, carbon n=34~38, double bonds=2~4; for CEs, carbon n=18, double bonds=1~3; SM (36:2;3) | 0.99 (-2.50,4.47) | 0.64 | -0.47 (-3.32,2.39) | 0.74 | 1.04 (0.91,1.18) | 0.692 | 1.01 (0.89,1.15) | 0.9 |
| **ME****magenta** | **15 (10 PCs, 2 LPCs, 2 SMs, 1PE)** | **For PCs, carbon n=30~36, double bonds=0~2; for LPCs, carbon n=16, 18, double bonds=1; for SMs, carbon n=34, 70, double bonds=1, 2; PE (16:0/16:1)** | **6.19 (3.02,9.35)** | **<0.001** | **1.92 (-0.96,4.79)** | **0.42** | **1.24 (1.1,1.39)** | **0.004** | **1.11 (0.98,1.27)** | **0.44** |

Model 1: adjusted for age, sex, region, and residence; Model 2: further adjusted for educational attainment, physical activity, current smoker, current drinker, body mass index,γ-glutamyl transpeptidase, creatinine, homeostatic model assessment of insulin resistance, total triglycerides, total cholesterol, hypertension, family history of chronic diseases, and medication status (antihypertensive or lipid-lowering medicines). CE, cholesteryl ester; Cer, ceramide; DAG, diacylglycerol; dhCer, dihydroceramide; GluCer, glucosylceramide; HexCer, hexosylceramide; LacCer, lactosylceramide; LPC, lysophosphatidylcholine; LPI, lysophosphatidylinositol; PC, phosphatidylcholine; PE, phosphatidylethanolamine, PE-O, alkylphosphatidylethanolamine, PE-P, alkenylphosphatidylethanolamine; PS, phosphatidylserine; SM, sphingomyelin; SM (OH), hydroxyl-sphingomyelin (with one additional hydroxyl); SM (2OH), hydroxyl-sphingomyelin (with two additional hydroxyls); TAG, triacylglycerol

Total population from the NHAPC study (n=3289) (n=3,289)

Excluded:

Participants without lipidomic data (n=1041)

Participant without uric acid data (n=1)

Population included in the current study (n= 2247)

Weighted gene co-expression network analysis identified modules consisting of biologically correlated lipids

Associations of individual lipids/ lipid modules with uric acid concentrations

Plasma lipidomic profiles quantified by liquid chromatography**-**electrospray ionization mass spectrometry

Associations between food groups and uric acid /hyperuricemia and related lipidomic biomarkers

Associations of individual lipids/ lipid modules with hyperuricemia risks

# Fig. S1 Flowchart of the study process


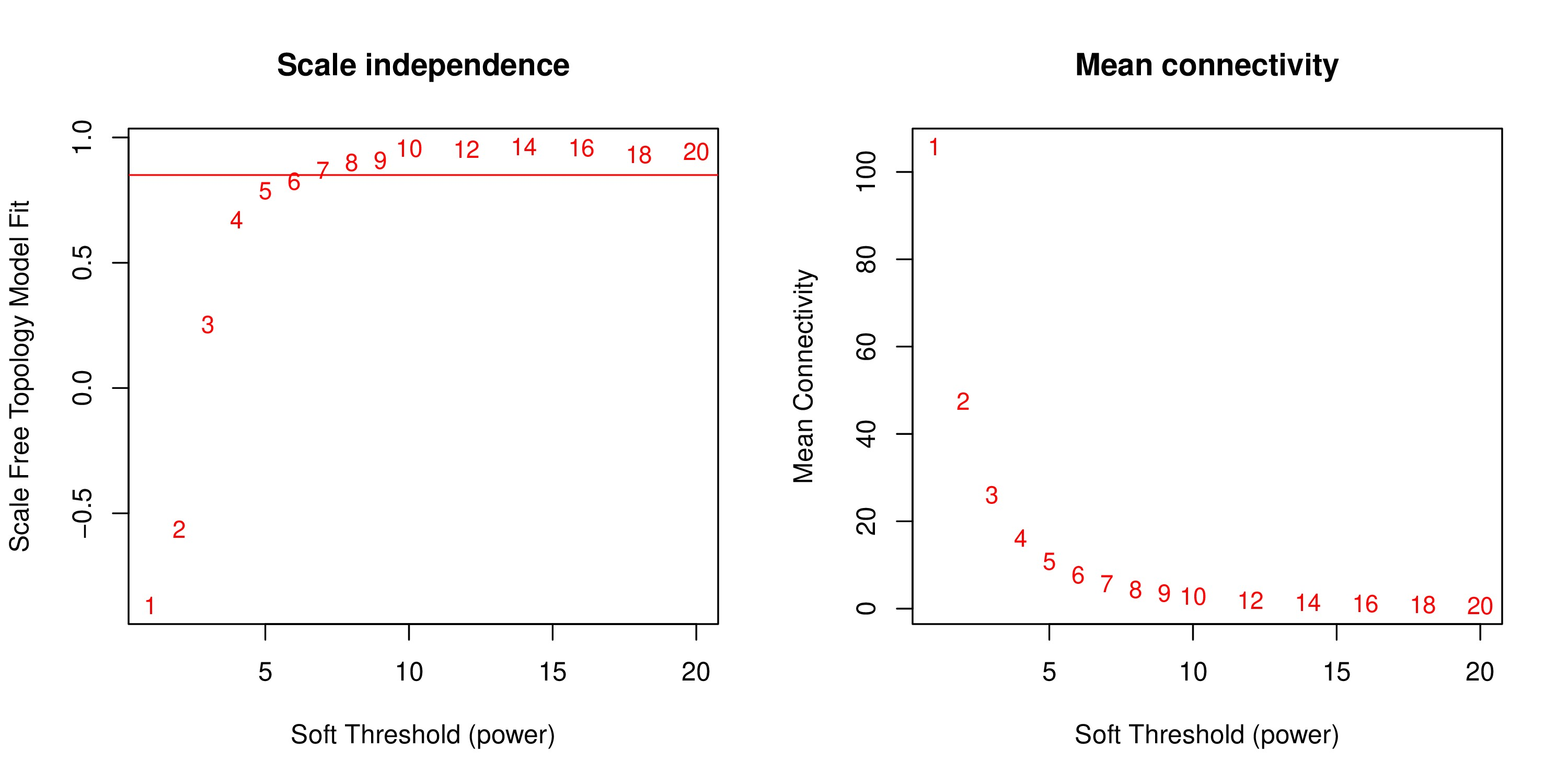


# Fig. S2 Analysis of network topology for various soft-thresholding powers. The smallest soft power (seven) with R^2^≥0.80 was chosen for the scale-free topology parameter in weighted correlation network analysis. The left panel showed the scale-free fit index (y-axis) as a function of the soft-thresholding power (x-axis). The right panel displayed the mean connectivity (degree, y-axis) as a function of the soft-thresholding power (x-axis)


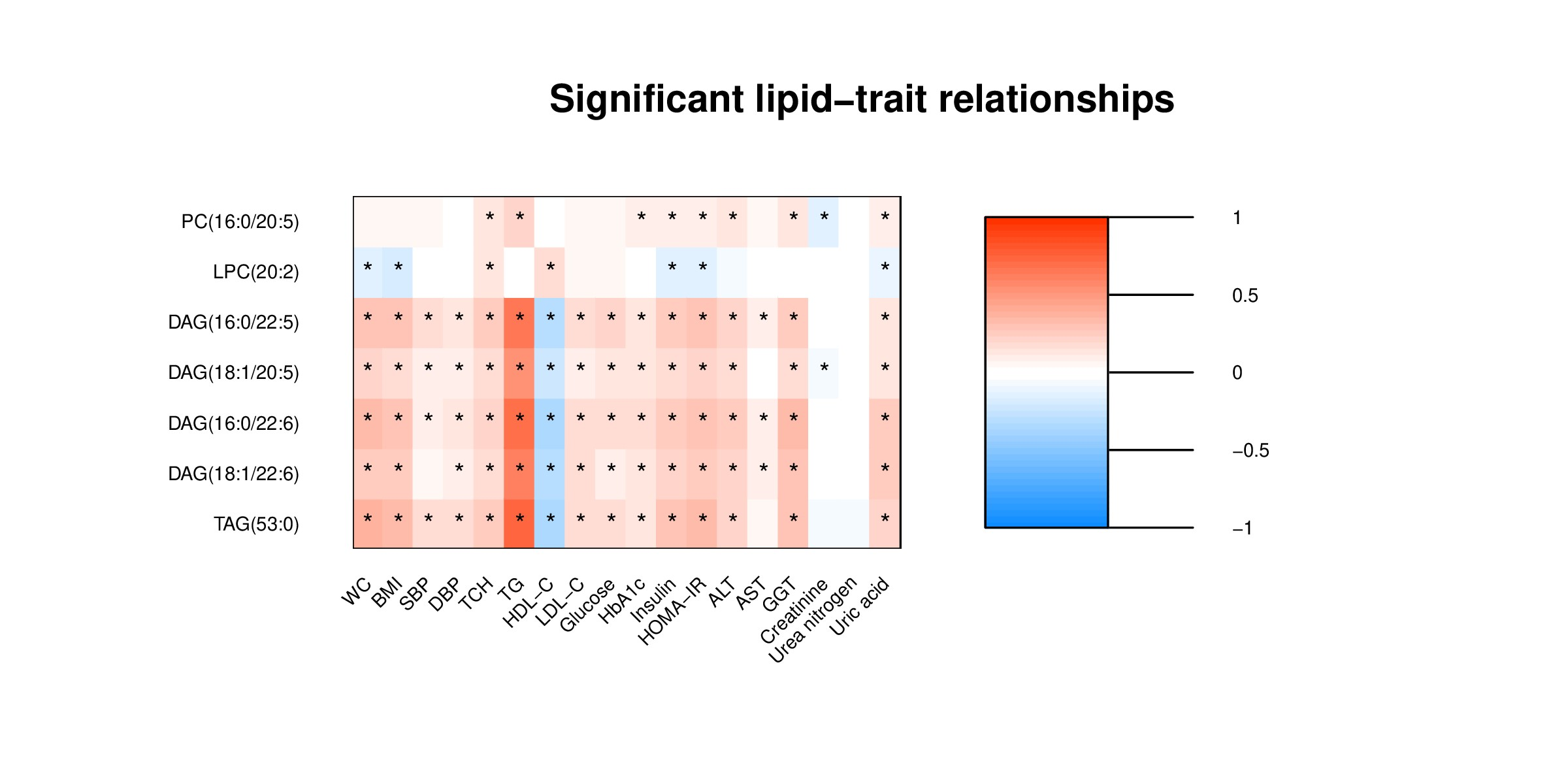


# Fig. S3 Correlation of lipids with metabolic traits. Only top significant lipids associated with HUA were showed in the graph. * indicates *p*< 0.05/(7*18) = 0.0004. Abbreviations: ALT, alanine aminotransferase; AST, aspartate transaminase; BMI, body mass index; DAG, diacylglycerol; DBP, diastolic blood pressure; eGFR, estimated glomerular filtration rate; GGT, γ-glutamyl transpeptidase; Glu, fasting glucose; HDL-C, high density lipoprotein cholesterol; HOMA-IR homeostatic model assessment of insulin resistance; LDL-C, low density lipoprotein cholesterol; LPC, lysophosphatidylcholine; PC, phosphatidylcholine; SBP, systolic blood pressure; TAG, triacylglycerol; TCH, total cholesterol; TG, total triglycerides; WC, waist circumference


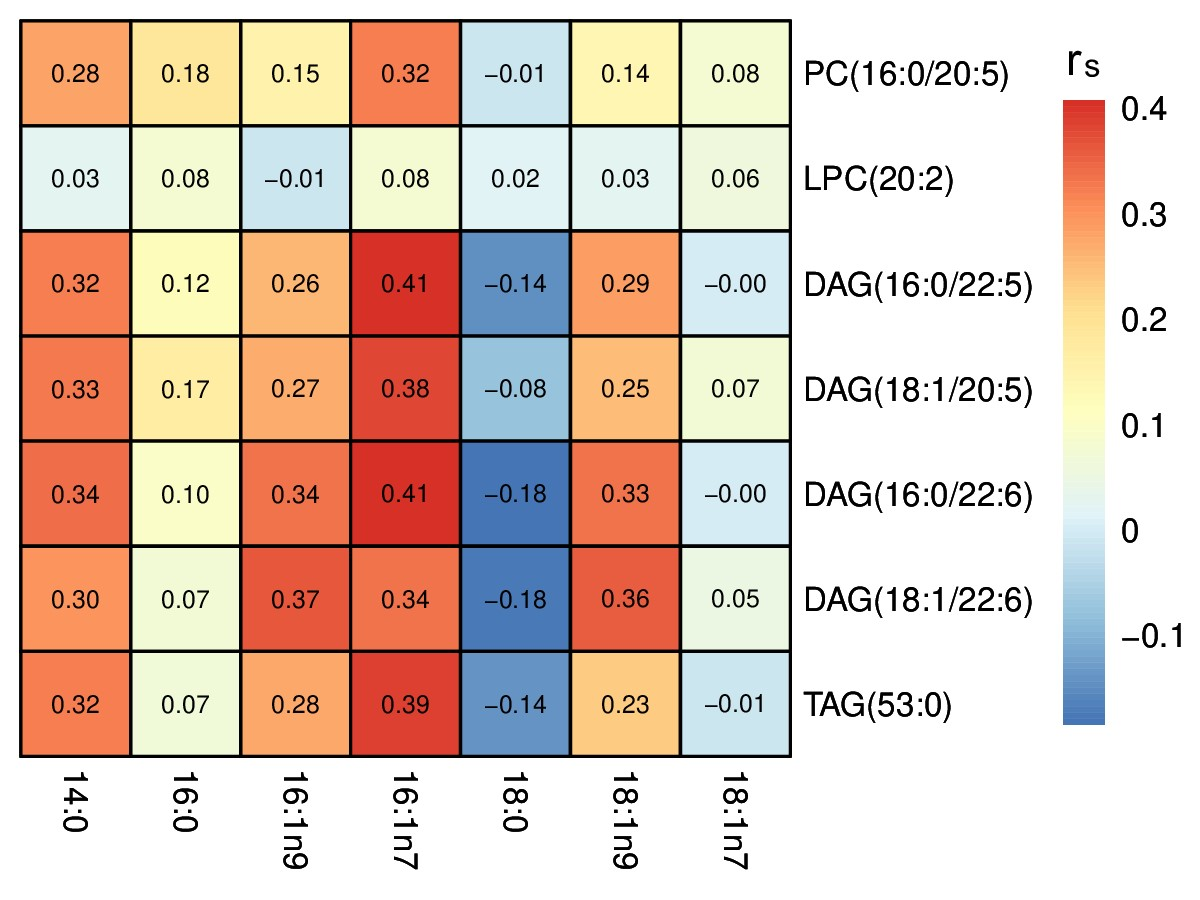


# Fig. S4 The Spearman correlations of lipids with fatty acids in the *de novo* lipogenesis pathway. Only top significant lipids associated with HUA were showed in the graph. DAG, diacylglycerol; LPC, lysophosphatidylcholine; PC, phosphatidylcholine; TAG, triacylglycerol; r_s_, Spearman correlation coefficients


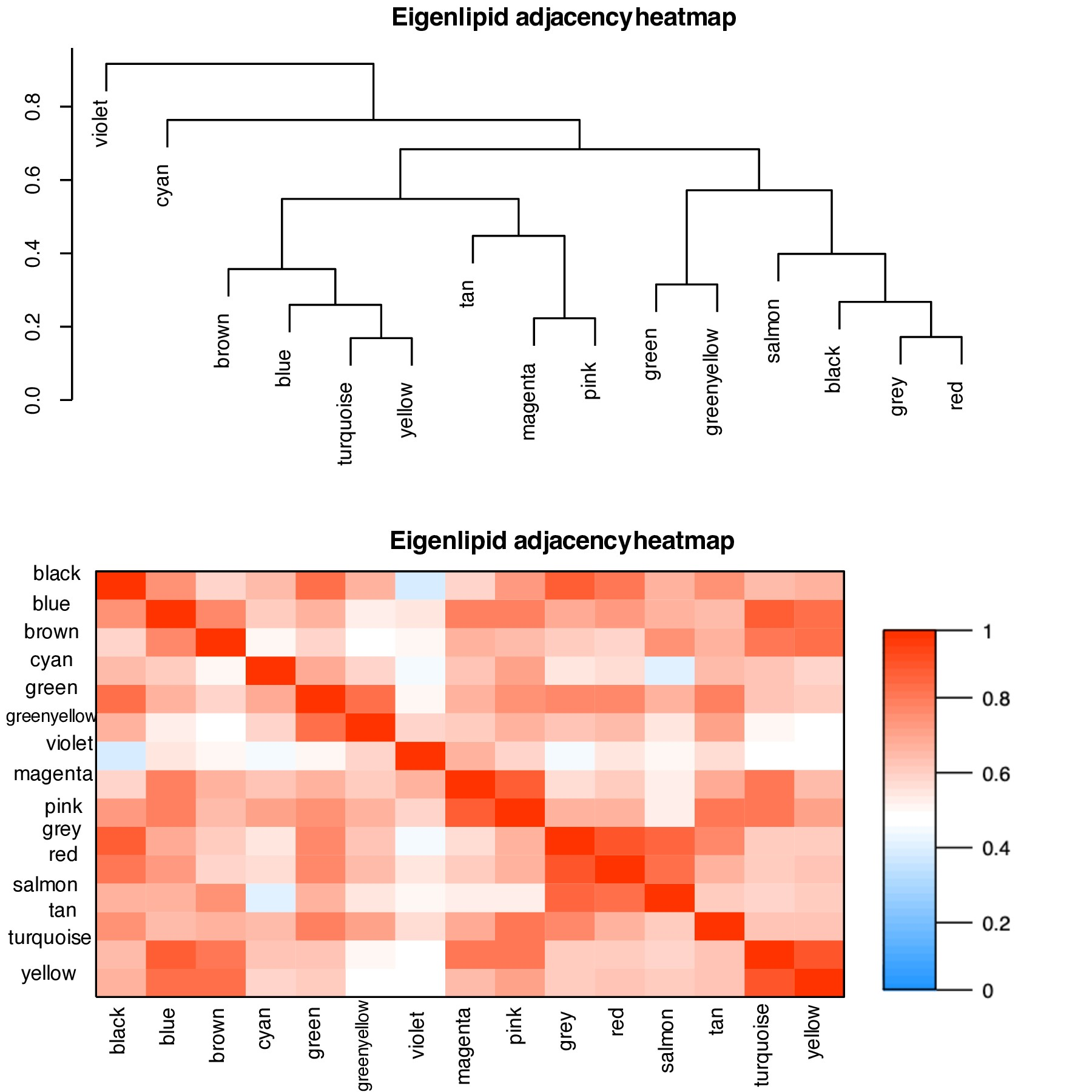


# Fig. S5 Module eigenlipid adjacency heatmap. The plot on the top was the cluster dendrogram of 15 modules after weighted correlation network analysis (WGCNA) analysis. Module-eigenlipid in this analysis were defined as the first principal component of a module matrix. The plot on the bottom was the heatmap showing the correlation of the 15 lipid modules identified by WGCNA, with red color representing higher correlation and blue color representing lower correlation


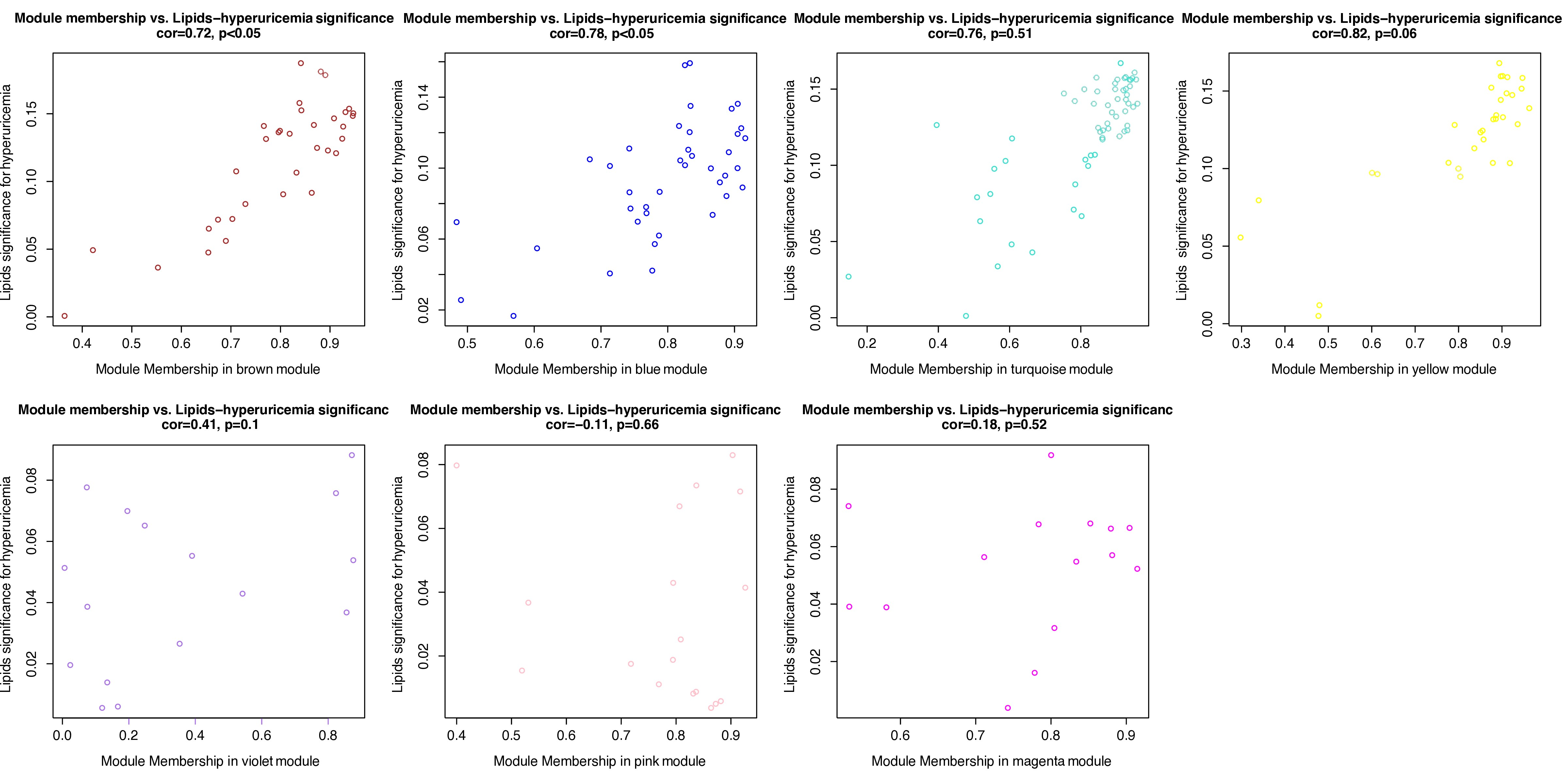


# Fig. S6 Scatterplots of Module Membership (MM) (x axis) vs. Lipid-hyperuricemia Correlation (y axis) in each cluster module. Module membership was calculated as the correlation between the lipid abundance and the module eigenlipid, representing how a specific lipid was connected to biologically interesting modules. There were significant correlations between MM and lipid-hyperuricemia correlations in brown and blue modules
